# Ion Channels in Pulmonary Hypertension: A Therapeutic Interest?

**DOI:** 10.3390/ijms19103162

**Published:** 2018-10-14

**Authors:** Mélanie Lambert, Véronique Capuano, Andrea Olschewski, Jessica Sabourin, Chandran Nagaraj, Barbara Girerd, Jason Weatherald, Marc Humbert, Fabrice Antigny

**Affiliations:** 1 Univ. Paris-Sud, Faculté de Médecine, 94270 Kremlin-Bicêtre, France; melanie.lambert91@hotmail.fr (M.L.); veronique.capuano@u-psud.fr (V.C.); barbara.girerd19@gmail.com (B.G.); jweatherald@gmail.com (J.W.); mjc.humbert@gmail.com (M.H.); 2 AP-HP, Centre de Référence de l’Hypertension Pulmonaire Sévère, Département Hospitalo-Universitaire (DHU) Thorax Innovation, Service de Pneumologie et Réanimation Respiratoire, Hôpital de Bicêtre, 94270 Le Kremlin-Bicêtre, France; 3 UMRS 999, INSERM and Univ. Paris–Sud, Laboratoire d’Excellence (LabEx) en Recherche sur le Médicament et l’Innovation Thérapeutique (LERMIT), Hôpital-Marie-Lannelongue, 92350 Le Plessis Robinson, France; 4 Ludwig Boltzmann Institute for Lung Vascular Research, Stiftingtalstrasse 24, Graz 8010, Austria; andrea.olschewski@lvr.lbg.ac.at (A.O.); nagaraj.chandran@lvr.lbg.ac.at (C.N.); 5 Department of Physiology, Medical University Graz, Neue Stiftingtalstraße 6, Graz 8010, Austria; 6 Signalisation et Physiopathologie Cardiovasculaire, UMRS 1180, Univ. Paris-Sud, INSERM, Université Paris-Saclay, 92296 Châtenay-Malabry, France; jessica.sabourin@u-psud.fr; 7 Division of Respirology, Department of Medicine, University of Calgary, Calgary, AB T1Y 6J4, Canada; 8 Libin Cardiovascular Institute of Alberta, University of Calgary, Calgary, AB T1Y 6J4, Canada

**Keywords:** PAH, TRPC, Orai, K^+^ channels, KCNK3, TMEM16A, Na^+^ channels, pulmonary hypertension

## Abstract

Pulmonary arterial hypertension (PAH) is a multifactorial and severe disease without curative therapies. PAH pathobiology involves altered pulmonary arterial tone, endothelial dysfunction, distal pulmonary vessel remodeling, and inflammation, which could all depend on ion channel activities (K^+^, Ca^2+^, Na^+^ and Cl^−^). This review focuses on ion channels in the pulmonary vasculature and discusses their pathophysiological contribution to PAH as well as their therapeutic potential in PAH.

## 1. Introduction

Pulmonary arterial hypertension (PAH) is a cause of severe pulmonary hypertension (PH) due to structural and functional alterations of the pulmonary circulation, leading to major remodeling of small pulmonary arteries (PA) less than 500 μm in diameter. This results in an abnormal elevation of mean pulmonary arterial pressure (PAP) above 25 mmHg, as compared to normal values of 14 ± 3 mmHg [1,2]. If untreated, this condition relentlessly progresses to right heart failure and ultimately death, in absence of lung transplantation. Increased pulmonary vascular resistance involves endothelial dysfunction, excessive proliferation of the pulmonary vascular smooth muscle and endothelial cells (ECs), inflammation and in situ thrombosis [2]. EC dysfunction is associated with increased cellular proliferation related to resistance to apoptosis, which contributes to pulmonary arterial obstruction. Associated with this, there is a decrease in the production of vasodilatory factors (nitric oxide (NO) and prostacyclin) and concurrent increase in the production of vasoconstrictor factors (endothelin 1 (ET-1) and angiotensin), growth factors (Platelet Derived Growth Factors (PDGF) and Fribloblast Growth Factor (FGF)) and pro-inflammatory factors (Interleukin-6 (IL-6) and Monocyte chemoattractant protein 1 (MCP1)) [3]. ECs also seem to play a key role in the endothelial–mesenchymal transition (EndoMT) which leads to plexiform and intimal lesions [4,5]. The excessive proliferation of pulmonary arterial smooth muscle cells (PASMC) can be partially explained by the imbalance between vasoconstricting and vasodilatory factors as well as angioproliferative factors produced by ECs [2]. Impressive advances in the understanding of the PAH pathobiology have been made regarding the mechanisms underlying proliferative and anti-apoptotic phenotypes and pulmonary vascular tone.

Genetics could partly explain these abnormal phenotypes of pulmonary vascular cells. Indeed, since 2000, several mutations have been identified in the Transforming Growth Factor-β (TGF-β) signaling pathway [6,7] and in *CAV1 (Caveolin-1)* and *TBX4 (T-Box 4)* genes [8,9]. More recently, in a large PAH cohort (1048 PAH patients), three new predisposing genes were identified: *ATP13A3*, *AQP1* and *SOX17* genes (ATPase 13A3, Aquaporine 1 and SRY-Box 17 [10]. These mutations constitute susceptibility factors for the development of PAH disease but are neither necessary nor sufficient.

In 2013, six different mutations were identified in the *KCNK3* gene (Potassium Channel, Subfamily K, Member 3) in PAH patients. Heterozygous *KCNK3* mutations are observed in sporadic and familial PAH patients, in which mutations segregate with the disease. *KCNK3* gene encodes for a potassium channel also called TASK-1 (TWIK-Related Acid-Sensitive K^+^ Channel 1). Patch-clamp experiments demonstrated a loss of function in all identified mutations [11,12]. More recently, two additional *KCNK3* mutations have been identified in Spanish cohort of PAH patients. Interestingly, this report described the first case of PAH occurring in a patient with homozygous *KCNK3* mutations associated with an aggressive form of PAH diagnosed at birth [13]. In addition, in 2017, two additional *KCNK3* mutations were identified in an American cohort of PAH patients [14]. To date, ten different *KCNK3* mutations have been described in PAH patients [11,13,15].

Moreover, single-nucleotide polymorphisms (SNPs) are found in the *KCNA5* gene (K_v_1.5, Potassium Voltage-Gated Channel Subfamily A Member 5) [16,17] and the *TRPC6* (for Transient Receptor Potential Canonical 6) gene promoter may predispose individuals to an increased risk of IPAH [18] but, to date, *KCNK3* mutations are the first channelopathies known to cause PAH [11]. Identified mutations or polymorphisms in ion channels demonstrate their crucial role in PAH. Indeed, ion channels, exchangers and pumps play an important role both in the systemic and the pulmonary vasculature, by controlling Ca^2+^ homeostasis, vascular tone, angiogenesis, cellular proliferation/apoptosis and diverse cellular processes.

Since the first therapeutic approach in the 1950s, treatments have evolved and, currently, PAH treatments target three main deregulated pathways: the ET-1 pathway with ET-1 receptor antagonist (Bosentan, Ambrisentan, and Macitentan), the NO pathway with PDE5 inhibitors (Sildenafil and Tadalafil) or guanylate cyclase stimulators (Riociguat) and the prostacyclin pathway with prostacyclin analogs (Epoprostenol and Treprostinil) or prostacyclin receptor agonist (Selexipag) [19].

Whereas major advances have been made in the PAH pharmacotherapy, many patients remain functionally impaired despite maximal medical therapy. This is why six new drugs targeting novel signaling pathways are currently under clinical trials, including drugs that target mitochondrial dysfunction (bardoxolone methyl), inflammation and immune dysfunction (Ubenimex, Rituximab, and Tocilizumab), iron deficiency (intravenous iron replacement), and oxidative stress (Dimethyl fumarate) [19].

Despite effective current therapies, no cure exists for PAH and lung transplantation remains the last treatment option for eligible PAH patients, with a 29% survival rate at 10 years post-transplantation [19]. Consequently, new therapeutic targets are needed to stop or minimize the development of this devastating disease and ion channels could be interesting targets for future PAH therapies. In the present review, we report and classify altered expression, function, and regulation of ion channels (K^+^, Ca^2+^, Na^+^ and Cl^−^ channels). We focus on the potential pathogenic role of ion channel deregulation in the onset and progression of PAH and their potential therapeutic interest.

### 1.1. Ion Channels in Pulmonary Vasculature

The pulmonary circulation is a closed circuit between the right side of heart (right ventricle, RV) and the lungs, as compared with the systemic circulation, which connect the heart and all other tissues. The RV is designed to pump blood through a low pressure and high-flow pulmonary vascular system. Due to the opposed functions of lung vessels (collecting oxygen) and systemic vessels (distributing oxygen), they developed different responses to hypoxia/hypoxemia. The pulmonary and systemic circulation have significant anatomical differences: arterioles of the systemic circulation have a dense layer of smooth muscle cells, whereas no such cells exist in the pulmonary vasculature.

Pulmonary arterial tone is mainly regulated by the resting membrane potential of PASMC and via pulmonary arterial endothelial functions. Resting membrane potential, intracellular ion homeostasis and cell volume are controlled by membrane permeability to cations and anions. Ion channels are ubiquitous and are constitutively active or modulated by several different stimuli such as signaling molecules or membrane potential changes. They are classified into two major classes:

- The cation channels, which comprise Na^+^, Ca^2+^, and K^+^ channels
- The anion channels, which include Cl^−^ channels and bicarbonate channels

The present review focuses on physiological and pathophysiological roles of Na^+^, Ca^2+^, K^+^, and Cl^−^ channels in the context of PH.

### 1.2. Role of Ion Channels in Pulmonary Arterial Tone

Pulmonary arterial constriction is initiated by an increase in intracellular Ca^2+^ concentration ([Ca^2+^]*_i_*) in PASMCs, while sustained vasoconstriction is maintained by both Ca^2+^-dependent and Ca^2+^-independent mechanisms. Pulmonary arterial constriction and dilatation are also regulated by factors released from pulmonary arterial endothelial cells (PAEC). Activity and expression of these factors, including endothelial nitric oxide synthase (eNOS), are required for synthesis at least in part depending on changes of [Ca^2+^]*_i_* in PAEC. A rise of [Ca^2+^]*_i_* in PAEC and PASMC has opposite effects on vascular tone. Indirectly, potassium (K^+^) conductance contributes to the regulation of plasma membrane potential [20], which participates in regulation of [Ca^2+^], and, consequently, of excitation–contraction coupling in pulmonary vascular smooth muscle. The membrane potential of PASMC is an important control mechanism of pulmonary arterial tone and hence arterial diameter.

**Figure 1:**
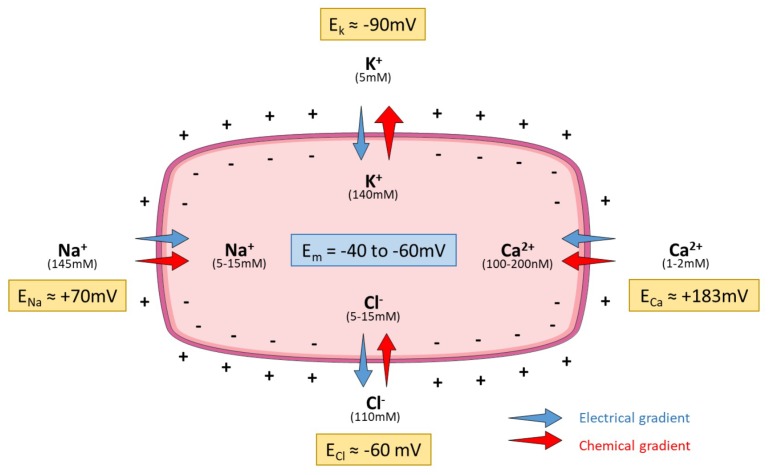
Concentration of the main ions (K^+^, Ca^2+^, Na^+^, Cl^−^) in mammalian cell as well as their electrical and chemical gradients.

Almost all mammalian cells, including PAEC and PASMC, have a negative resting membrane potential close to the equilibrium potential for K^+^ ions (*E*_k_). The electrical difference between the cytosol and the extracellular space ranges from −85 to −60 mV in excitable cells (including PASMC) and from −55 to −30 mV in non-excitable cells (including PAEC). In vitro, the resting membrane potential of PASMC varies from −65 to −50 mV, close to the theoretical *E_k_* (−90 mV) [21]. High intracellular K^+^ (140mM) and low extracellular K^+^ (5mM) concentrations are mostly maintained by the Na^+^/K^+^ pump, while the opening of K^+^ channels hyperpolarizes the plasma membrane (Figure 1, based on [22]).

### 1.3. Current Methods to Measure Ion Channels Activities

**Figure 2:**
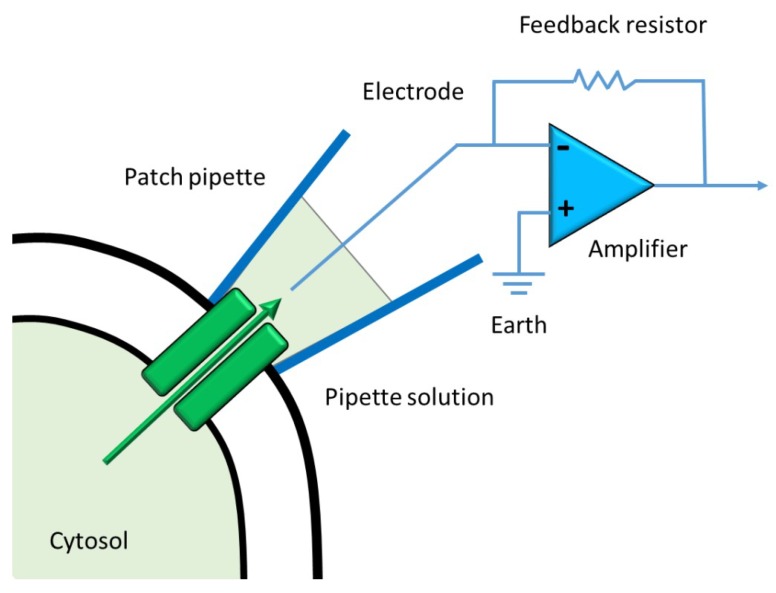
General principle of patch-clamp recording. A glass pipette containing electrolyte solution is seeded onto the cell membrane, isolating a membrane patch electrically. Currents are recorded by an electrode connected to highly sensitive amplifier.

**Figure 3:**
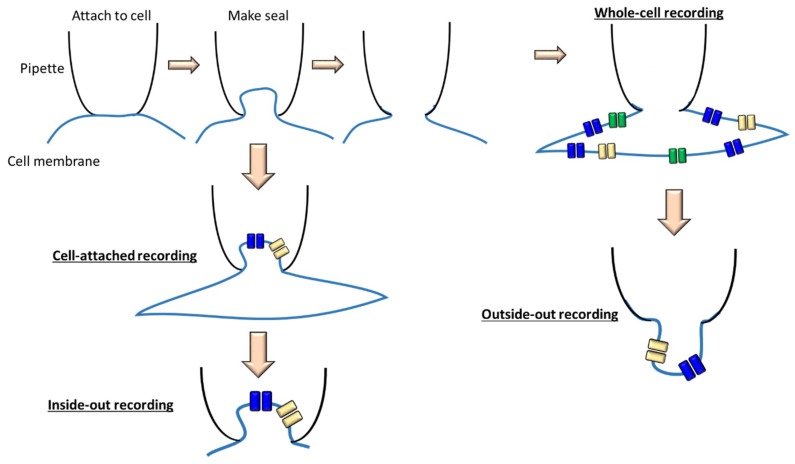
Patch-clamp electrophysiology. Formation of the giga seal and the subsequent cell-attached and whole-cell configuration. When the pipette is in closest proximity to the cell membrane, mild suction is applied to gain a tight seal between the pipette and the membrane. Whole-cell: By applying another brief but strong suction, the cell membrane is ruptured and the pipette gains access to the cytoplasm. Inside-out: In the cell-attached mode, the pipette is retracted and the patch is separated from the rest of the membrane and exposed to air. The cytosolic surface of the membrane is exposed. Outside-out: In the whole-cell mode, the pipette is retracted resulting in two small pieces of membrane that reconnect and form a small vesicular structure with the cytosolic side facing the pipette solution.

Ion channels are selectively permeable to specific ions and the best technical approach to measure the activity of each ion channel is the patch-clamp technique. Patch-clamping records the electrical activity of a microscopic fragment of cell membrane, electrically isolated from the rest of the cell surface. We use a glass electrode (1 µm diameter) filled with a conductive solution (Figure 2). The electrode is manipulated using a micromanipulator, moved from the cell surface, and then “sealed” on the membrane by imposing a depression inside the glass capillary via a connected catheter to an air intake on the electrode holder. When the orifice of the pipette is sealed on an intact cell, this configuration is called “attached” (Figure 3). In the cell-attached patch, unitary currents are produced by ion flux through single channels.

A second configuration (Figure 3) is obtained by separating the pipette and patch from the cell. During this procedure, the membrane patch remains attached to the pipette and its intracellular face, directed towards the outside of the pipette (Inside-out configuration).

A third configuration is called “whole cell recording or whole cell configuration”. Starting from the “Cell-attached” configuration, we break the patch by exerting a strong suction and the pipette solution enters into contact with the intracellular medium. In the whole-cell mode, the macroscopic current recorded is the summation of all the currents generated by similar channels throughout the cell. We do not distinguish individual channels (Figure 3). Macroscopic currents are characterized by the current amplitude, activation, inactivation and deactivation during a pulse protocol. In the whole-cell mode, the pipette is retracted resulting in two small pieces of membrane that reconnect and form a small vesicular structure with the cytosolic side facing the pipette solution (Figure 3). These configurations make it possible to study the ion channels at the individual level (single channel). Single-channel recordings can provide the amplitude of unitary currents, the open probability of the channels and the amount of time the channel(s) spend in open (open duration) or closed (closed duration) conformations.

Using whole cell configuration in current clamp mode (*I* = 0), resting membrane potential could be evaluated using membrane potential indicators (Potential-sensitive ANEP dyes, DiBAC4, and FluoVolt membrane potential dye).

The patch-clamp technique allows us to record all ion channel families (K^+^, Ca^2+^, Na^+^, and Cl^−^); however, an alternative strategy can be used to measure Ca^2+^ channel function via Ca^2+^ imaging. Ca^2+^ imaging is a method for monitoring changes of intracellular free calcium concentrations in cells or tissues taking advantage of fluorescent indicators, including small chemical indicators (Fura-2, Fluo-4, Fluo-3, and Indo-1) and genetically encoded calcium indicators that change spectral properties upon binding calcium.

### 1.4. Preclinical Animal Model of PH

Most of our understanding of the molecular mechanisms responsible for PH is derived from rodent PH models. The three most used rodent models are:

(i) Monocrotaline-induced PH Model: Monocrotaline (MCT) is derived from the plant *Crotalaria spectabilis*. CYP3A4-dependent (Cytochrome P450 3A4) hepatic metabolism creates a reactive MCT pyrrole. A single dose of MCT (40–100 mg/kg, subcutaneously) causes PH in rats within 3–4 weeks and leads to death due to RV failure. The MCT-PH rat model recapitulates many features of human PAH, such as early endothelial injury, adverse pulmonary vascular remodeling and mitochondrial metabolic abnormalities in the pulmonary vasculature and RV [23].

(ii) Chronic Hypoxia (CH) Model: Exposing rodents (mice or rats) to 10% oxygen for 3–4 weeks in normobaric or hypobaric hypoxia induces pulmonary arterial medial hypertrophy, elevated mPAP and RV hypertrophy [24]. The CH model does not cause obstructive pulmonary arterial intimal lesions and induces only mild PH [24]. Finally, PH and pulmonary vascular remodeling reverse when the animals return to normoxia.

(iii) Sugen 5416-Hypoxia Model (SU/Hx): After a single subcutaneous (20 mg/kg) injection of the vascular endothelial growth factor receptor blocker Sugen 5416 (SU), rats are exposed to hypoxia (10% oxygen) for three weeks. Thereafter, rats return to normoxia for an additional 5–11 weeks. SU/Hx animals develop plexiform lesions that mimic the histology observed in human PAH, however, SU/Hx exposition does not lead to RV failure as observed MCT-PH model [23,25].

## 2. Functional and Molecular Classification of Ion Channels in the Pulmonary Circulation

### 2.1. K^+^ Channels

To date, eight families of K^+^ channel genes have been cloned and identified, and each of these families covers a large number of individual members; more than 60 different subunits have been identified. In the pulmonary vasculature, numerous K^+^ channels are functionally expressed including voltage-gated K^+^ channels (K_v_), Ca^2+^-activated K^+^ channel (K_Ca_), inward rectifier K^+^ channel (K_ir_), ATP-sensitive K^+^ channel (K_ATP_) and two-pore domain K^+^ channels (K_2P_). Each family needs to tetramerize or heterotetramerize to form a functional channel, increasing the diversity of channels.

#### 2.1.1. Inward Rectifier Channel (K_ir_) Family

There are seven K_ir_ channel subfamilies (K_ir_1–7) [26]. K_ir_ channels only have two transmembrane domains. They do not have intrinsic gating mechanisms and therefore rely on interaction with cytosolic cations (Mg^2+^) and polyamines to regulate their opening [27].

In smooth muscle, currents through K_ir_ channels have so far only been described in coronary arterial smooth muscle and may be preferentially expressed in small rather than large arteries [28]. Classical K_ir_ channels, principally K_ir_2.2, are present in ECs and smooth muscle cells and play a key role in vascular tone. In addition, K_ir_2.1 promotes dilatation of rat coronary and cerebral arteries in response to an increase in the external K^+^ concentration.

These K^+^ channels are thought to be important in maintaining EC resting membrane potentials [29]. In ECs, by setting a negative resting potential, they enable Ca^2+^ entry in the cell and activate production of NO [30].

#### 2.1.2. ATP-Sensitive K^+^ Channel (K_ATP_)

K_ATP_ channels are composed of four K_ir_6.*x* channels and four sulfonylurea receptor subunits (SUR1, SUR2A and SUR2B). SUR1 encoded by the *ABCC8* gene, and *SUR2A* and *SUR2B*, which are splice variants arising from a single ABCC9 gene. The K_ir_ subunits form the pore and are responsible for the ATP channel inhibition, whereas the SUR subunits that contain two nucleotide-binding domains bind nucleotide diphosphates (Nucleoside-diphosphate (NDPs); e.g., Adenosine-diphosphate (ADP)) and activate K_ATP_ channels. K_ATP_ channels open spontaneously, openings that are inhibited by intracellular ATP via interaction with K_ir_ subunits [31], whereas channel activation by Mg^2+^-dinucleotides occurs via interaction with the SUR subunits. K_ATP_ channels are expressed in the cardiovascular system and especially in the pulmonary circulation [32].

##### Pulmonary Arterial Smooth Muscle Cells

In the vascular smooth muscle cells, K_ATP_ channels have numerous roles. More specifically, in the PASMC, K_ATP_ channels contribute to the maintenance of membrane potential (*E*_m_). In addition, the closure of the K_ATP_ channels could lead to an increase in Ca^2+^ concentration into the cytoplasm which would enhance contraction and proliferation of PASMCs [33]. In contrast, opening of K_ATP_ channels causes an increase in K^+^ efflux, membrane hyperpolarization, inhibition of Ca^2+^ influx, and relaxation of the PASMC. Finally, K_ATP_ may also be activated by the NO and the endothelium-derived hyperpolarizing factor (EDHF) [34].

##### Pulmonary Arterial Endothelial Cells

In the endothelium, the role of K_ATP_ is less studied. However, K_ATP_ channels in pulmonary ECs are responsible for maintaining resting membrane potential and a decrease of shear stress inactivate K_ATP_ channels leading to plasma membrane depolarization [35].

#### 2.1.3. Voltage-Gated K^+^ Channels (K_v_)

K_v_ channels are composed of four pore-forming α subunits, which are composed of six transmembrane domains (S1–S6) associated with an ancillary β subunit [36]. To be fully functional, they have to be in a tetrameric organization [37]. Each monomer is formed by the pore domain and the voltage sensor domain [38]. Each α subunit can be associated with β subunits, which determines the characteristics of the channel (inactivation and deactivation kinetics, and voltage sensitivity). As their name suggests, their activation depends on the voltage of the membrane and they have a high selectivity for K^+^ [39].

The expression and proper functioning of K^+^ channels in PASMC are essential to the regulation of vascular tone and cell viability under pathological conditions [20]. Dysfunction and downregulation of K^+^ channels play an important role in the development of pulmonary vasoconstriction and vascular remodeling in patients and animals [40]. Indeed, an increased concentration of intracellular K^+^ inhibits caspase activity which induces a resistance to apoptosis of the SMC [41]. In addition, the increase of the concentration of intracellular K^+^ ions in the cell induces a depolarization of the cell membrane associated with an elevated intracellular Ca^2+^ concentration, stimulating SMC proliferation [42].

##### Pulmonary Smooth Muscle Cells

In human PASMCs, at least 22 voltage-gated channels subtypes are expressed at mRNA levels as reported in the listing below [27,43,44]. Archer et al. reported the presence of K_v_1.1, -1.2, -1.3, -1.5, -1.6, and -2.1, but they failed to detect K_v_1.4 [43]. At the same time, Yuan et al. demonstrated the expression of K_v_1.1, -1.2, -1.4, -1.5, -1.6, -2.1, and -9.3, but not K_v_1.3 [44]. A few years later, expression of the K_v_3.1 subtype was reported by other groups [27,45]. Although other subtypes (K_v_1.7, -4.1, and -9.2) have also been identified [46], K_v_1.1, -1.2, -1.5, -2.1, -9.3, and -3.1 subtypes are expressed in PASMCs, and one (or a co-assembly) of these types may act as oxygen sensors [47]. To date, four types of β subunits (K_v_β1–4) have been identified in association with the pore-forming α-subunit. Among these types, K_v_β1, -β2, and -β3 subunits are expressed in PASMCs [48]. Although the exact function of β subunits is not known, β subunits seems to play a role as a redox sensor and in adaptation to hypoxia [49]. Downregulation of K_v_1.5 expression using antisense oligonucleotides or short interfering RNA (siRNA) causes membrane depolarization and increases cytosolic Ca^2+^ concentration. Pharmacological inhibition of K_v_ channels by 4-Aminopurydine (4-AP) and correolide (K_v_1.3 inhibitor) stimulate PAMSC contraction, migration [50], and proliferation [51], but not apoptosis [52]. More recently, mRNA level expression of KCNQ4 (Potassium Voltage-Gated Channel Subfamily Q Member 4, K_v_7.4) was observed in rat pulmonary artery. KCNQ4 activators stimulate pulmonary arterial dilatation, suggesting that KCNQ4 isoform have an important role in the regulation of pulmonary arterial tone and could provide a novel treatment target in PAH [53].

##### Pulmonary Endothelial Cells

Immunostaining experiments revealed that rat PAEC express K_v_1.5 and K_ir_2.1 channels. Patch-clamp experiments performed on freshly isolated PAEC demonstrated different K^+^ current (*I*_K_) profiles. Indeed, in 90% of PAEC, *I*_Kv_ is identified and the remaining 10% possessed an *I*_Kir_ [54].

#### 2.1.4. Ca^2+^-Activated K^+^ Channels (K_Ca_)

K_Ca_ channels are comprised of six transmembrane segments and 1 pore domain (S0–S6), with a positively charged S4 segment, that create one pore in the membrane. There are five families of Ca^2+^-sensitive channels (K_Ca_1–K_Ca_5). They share a high degree of homology with the K_v_. K_Ca_ channels are activated by membrane depolarization and are deactivated by membrane hyperpolarization. A fundamental difference between K_v_ and K_Ca_ channels function is their response to intracellular Ca^2+^ variations. In vascular smooth muscle cells, K_v_ channels are inhibited by high cytoplasmic Ca^2+^ concentration and K_Ca_ channels are activated by cytosolic Ca^2+^ [55].

K_Ca_ channels are characterized by three different types; the Maxi-K channels (BK_Ca,_ with a conductance of 100–300 pS), the intermediate-conductance channels (I_KCa_, with a conductance of 25–100 pS) and the small-conductance channels (SK_Ca_, with a conductance of 2–25 pS) [56].

The BK_Ca_ channel is voltage and Ca^2+^ dependent. It activates at a membrane potential higher than 20 mV and at intracellular Ca^2+^ concentration above 300 nM. BK_Ca_ directly binds Ca^2+^ at its C-terminal region.

The activation of I_KCa_ channels is not dependent on voltage but on intracellular Ca^2+^ concentration [57,58]. This is conferred by CaM binding in the C-term end of the channel, in a similar way as for SK_Ca_.

The activation of SK_Ca_ channels is voltage independent and their current–voltage relationship shows an inward rectification [59]. At positive membrane potentials, Ca^2+^ not only activates but also blocks SK_Ca_ channels in a concentration-dependent manner [60]. Intracellular Ca^2+^ concentrations of 300–700 nM activate 50% of SK_Ca_ channels [61].

##### Pulmonary Smooth Muscle Cells

In human PASMC, at least five isoforms of Ca^2+^-activated potassium (K_Ca_) channels appear to be expressed at mRNA levels: Maxi-K_Ca_α1 and Maxi-K_Ca_β1–β4. K_Ca_ channels in vascular smooth muscle cells are targets for various physiological factors released from the endothelium, including NO [61] and EDHF [62].

##### Pulmonary Endothelial Cells

Immunoblotting and RT-PCR revealed the presence of BK_Ca_ channel α- and β-subunits in lung microvascular endothelial cells. Activation of endothelial BK_Ca_ channels causes hyperpolarization, which leads to an increase in NO production and subsequent endothelium-dependent vasodilation in pulmonary vasculature [63]. Immunohistochemistry and Western blot analysis demonstrated the expression of K_Ca_2.3 and K_Ca_3.1 channels in the endothelium of human pulmonary arteries. Using specific K_Ca_2.3 blockers (UCL1684), Kroigaard and collaborators demonstrated that these channels contribute to endothelium-dependent relaxation, indicating that K_ca_2.3 channels are a potential target for pulmonary hypertensive pathology [64]. Prostacyclin analogs, which are used in treatment of PH, are potent activators of K_Ca_ channels (patch clamp) through peroxisome proliferator-activated receptor-β/δ as a rapid signaling factor in human PASMC [65].

#### 2.1.5. Two Pore Potassium Channels (K_2P_)

**Figure 4:**
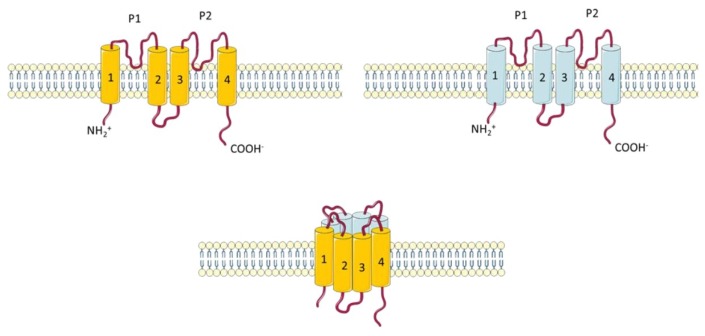
General molecular architecture of two pore potassium channels (K_2P_). K_2P_ channels need homodimerization or heterodimerization to form a functional channel (different colors represent different K2P subunit).

Two pore potassium channels, also called background conductance channels, mediate resting membrane potentials. Different K_2P_ units share only moderate sequence homology outside their pore region but they are characterized by the same general molecular architecture (Figure 4). These channels form a single pore by making a dimer of two subunits. They are unique in having four transmembrane domains and two pores to conduct K^+^ ions with high selectivity for K^+^. Their main function is to maintain the resting membrane potential [66]. They are known to be regulated by several mechanisms including oxygen tension, pH, mechanical stretch, and G-proteins [67].

The K_2P_ family is constituted by 15 members, divided into five families with different electrophysiological and/or pharmacological properties [68]:

TWIK = Tandem of P domains in Weak Inward rectifier K^+^ channel

TREK/TRAAK = TWIK-Related K^+^ channel/TWIK Related Arachidonic Acid-StimulatedK^+^ channel

THIK = Tandem pore domain Halothane-Inhibited K^+^ channel

TALK = TWIK-related Alkaline pH-Aactivated K^+^ channel

TASK = TWIK-related Acid-Sensitive K^+^ channel [69]

As represented in Table 1, the K_2P_ family (named KCNK channels for K^+^ Channel subfamily K) gather different sub-families which are composed of several members [70]. The TASK family includes TASK-1, -2, -3, -4 and -5, although TASK-5 does not seem to produce a functional channel when expressed in artificial systems. The TWIK, THIK and TALK families include two members designated by TWIK-1 and -2, THIK-1 and -2, TALK-1 and -2, respectively. Finally, the TREK family contains three members: TREK-1, TREK-2 and TRAAK (Table 1).

Gardener and collaborators demonstrated that rat pulmonary arteries (PA) expressed TASK-1, TASK-2, THIK-1, TRAAK, and TREK-1 at the mRNA level, and confirmed protein expression of TASK-1, TASK-2, TREK-1 and TWIK-2. Using micro-electrodes measurement on intact arteries, these authors suggested that the pharmacological modulation of TASK-1 (KNCK3) induced membrane potential shifting by approximately 10 mV in a hyperpolarizing or depolarizing direction, suggesting that KCNK3/TASK-1 function contribute to resting membrane potential [71].

##### Pulmonary Arterial Smooth Muscle Cells

There is a general agreement that the resting potential of PASMC is determined largely by a non-inactivating K^+^ conductance (*I*_KN_) that is sensitive to inhibition by 4-aminopyridine (at relatively high (1 mM) concentrations) [72]. The resting potential and *I*_KN_ display low sensitivity to the drugs quinine and Ba^2+^ ions and are insensitive to tetraethylammonium (TEA), glibenclamide, and a range of Ca^2+^-activated K^+^-channel blockers. Moreover, *I*_KN_ and the resting potential are enhanced by halothane (1 mM), inhibited by Zn^2+^ (100–200 μM) and anandamide (10 μM), and insensitive to cytoplasmic Ca^2+^. All these properties correspond to the properties of TASK-1/KCNK3 channel, which is highlighted in rabbit PASMC [73]. In humans, KCNK3/TASK-1/ is expressed in cultured PASMC. Moreover, in human PASMC, knockdown of KCNK3 (by siRNA strategy) induced a significant depolarization of resting membrane potential (−40 mV in cells treated with si-Control and −25 mV in cells treated with si-KCNK3), confirming the crucial implication of KCNK3/TASK-1 channels in the resting membrane potential [68]. An interesting study highlighted that 3–4 days in culture condition induced loss of K_v_1.5, KCNK3 and K_v_1.2 channels expression, emphasizing the importance of using freshly dissociated PASMC compared to cultured PASMC [74]. Unexpected results using double knockout TASK-1/TASK-3 mice demonstrated that KCNK3/TASK-1 channel is not functional in mice PASMC [75], excluding mice as model of study for KCNK3/TASK-1 channels in the pulmonary vasculature. It was suggested that overexpression of TASK-3 channels (not endogenously expressed in lung) might be able to compensate the absence of KCNK3/TASK-1 expression in mice [12].

##### Pulmonary Arterial Endothelial Cells

Immunolocalization experiments in intact PAs showed that KCNK3/TASK-1 and TASK-2 are prominent in the PASMC compared to the endothelium. TWIK-2 is also present in the smooth muscle cells but absent in the endothelium [71]. We also recently confirmed by immunolabeling experiments that KCNK3/TASK-1 is expressed in human and rat PAEC and functionally expressed in freshly isolated rat control PAEC (patch-clamp) [76].

### 2.2. Ca^2+^ Channels

**Figure 5:**
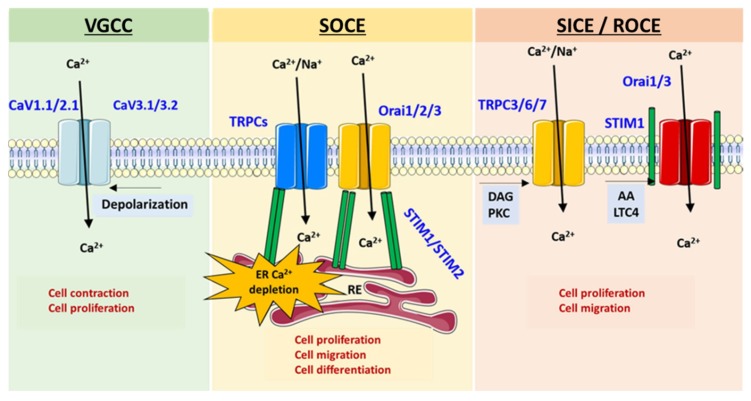
Coexistence of Voltage-Gated Ca^2+^ Channels (VGCC), non-voltage-dependent Store-Operated Ca^2+^ Entry (SOCE) and non-voltage-dependent Store-Independent Ca^2+^ Entry (SICE or ROCE) in mammalian cells.

Tight control of [Ca^2+^]*_i_* is crucial for cell survival and normal cell function. Cells must maintain resting [Ca^2+^]*_i_* at low level (around 100 nM) to create the wide dynamic range required for Ca^2+^ signals. This finely tuned control of [Ca^2+^]*_i_* is essential for differential modulation of various signaling pathways and intracellular Ca^2+^-regulated proteins, which in turn are involved in specific cellular processes such as the regulation of metabolism, gene transcription, cell proliferation, migration and death, exocytosis, and contraction [77]. Intracellular Ca^2+^ signaling is generated by Ca^2+^ release from the internal stores and/or by Ca^2+^ entry from the extracellular compartment through Ca^2+^ or cationic channels. In mammalian cells, three different Ca^2+^ influx co-exist (Figure 5):

(i) Voltage-Gated Ca^2+^ Channels (VGCC)
(ii) Non-voltage-dependent Store-Operated Ca^2+^ Entry (SOCE)
(iii) Non-voltage-dependent Store-Independent Ca^2+^ Entry (SICE) also called Receptor-Operated Ca^2+^ Entry (ROCE)

#### 2.2.1. Voltage-Gated Ca^2+^ Channels (VGCC)

VGCC are a group of voltage-gated ion channels expressed in the plasma membrane of all excitable cells as well as many types of non-excitable cells [78]. VGCC is activated by plasma membrane depolarization which leads to a rise in [Ca^2+^]*_i_*. VGCCs are found as six different types (L, T, N, P/Q and R types) based on functional characteristics (e.g., conductance, voltage-dependence, time-dependent kinetics of channel opening and closing, pharmacological properties, and cell/tissue distributions).

In PASMC, two main types of VGCC are expressed, the high voltage-activated (HVA) L-type and low voltage-activated (LVA) T-type channels that represent the major pathways for voltage-mediated Ca^2+^ entry involved in cell function such as excitation–contraction coupling [79], excitation–transcription coupling and cell proliferation [80,81,82].

**Figure 6:**
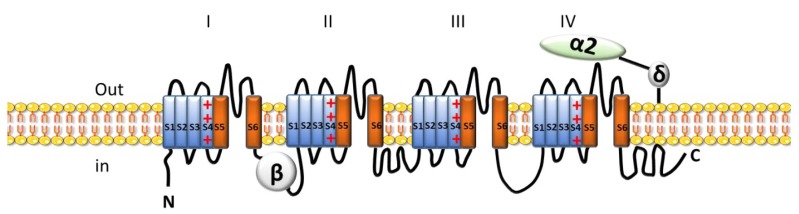
Schematic illustration of high voltage-activated (HVA) L-type Ca^2+^-channels. α subunit composed of the N terminus, the four homologous domains with six transmembrane segments, and a reentrant pore loop in each (I–IV), and the C-terminus.

The structure of HVA channels contains five subunits: α1, α2/δ, β and γ. The α1 subunit consists of four domains (DI–DIV) formed by six transmembrane α helices (S1–S6) and a membrane associated loop (between S5 and S6). The S4 segment of each domain serves as the voltage sensor. The S5 and S6 segments and the membrane-associated pore loop between them form the pore lining of the voltage-gated ion channels and the receptor site for pore-blocking (Figure 6). Unlike members of the HVA channels, LHA channels do not require auxiliary Ca^2+^ channel subunits. The inner pore is lined by the S6 segment, which forms the receptor sites for the pore-blocking Ca^2+^ antagonist drugs specific for L-type Ca^2+^ channels [83].

All Ca^2+^ channels share these general structural features but the amino acid residues that confer high affinity for the organic Ca^2+^ antagonists used in therapy of cardiovascular diseases are present only in the Ca_v_1 family of Ca^2+^ channels, which conduct L-type Ca^2+^ current.

L-type channels have a unitary conductance ranging from 20 to 27 pS using 110 mM Barium (Ba^2+^) as the charge carrier, whereas T-type channels have less conductance, from 5 to 8 pS. In comparison to L-type Ca^2+^ current (*I*_CaL_), the T-type current (*I*_CaT_) is activated at much more negative membrane potentials, inactivates rapidly, deactivates slowly and is insensitive to Ca^2+^ channel blockers (CCBs) 14-dihydropyridines (DHPs). Four isoforms of α1 L-type subunits are identified, namely *CACNA1S* (Ca_v_1.1), *CACANA1C* (Ca_v_1.2), *CACNA1D* (Ca_v_1.3 and *CACNA1F* (Ca_v_1.4), whereas three isoforms are described for T-type channels, *CACNA1G* (Ca_v_3.1), *CACNA1H* (Ca_v_3.2) and *CACANA1I* (Ca_v_3.3) [84,85].

##### Pulmonary Arterial Smooth Muscle Cells

In PASMC, the L-type channel, Ca_v_1.2 is widely accepted as the source for depolarization Ca^2+^ concentration [86] whereas T-type channels Ca_v_3.1 and Ca_v_3.2 are involved in cell proliferation [81,82]. Recent studies showed, by immunostaining and confocal imaging, that both Ca_v_3.1 and Ca_v_3.2 isoforms are revealed in human lung tissue and cultured PASMCs; Ca_v_3.1 labeling is mainly in the cytoplasm, whereas Ca_v_3.2 labeling is strongly localized in the nucleus [82]. As in the cardiomyocyte, the T-type signaling activates the phosphatase PP2A (Protein phaspatase 2) in PASMC, regulating the activities of kinases such as ERK1/2 (Extracellular signal-regulated kinases 1/2), P38 and Akt1 (or protein kinase B), which are downstream targets involved in cell proliferation and apoptosis [81,82].

##### Pulmonary Arterial Endothelial Cells

T-type channels are expressed in ECs in the lung microcirculation where they mediate vaso-occlusion of sickled erythrocytes [87], promote P-selectin surface expression [88] and control von Willebrand factor secretion [89]. Altogether, they are involved in the endothelial proinflammatory phenotype transition. Ca_v_3.1 channels are also expressed in intra-pulmonary arteries where they are involved in vasoconstriction and relaxation mediated by acetylcholine [90,91].

#### 2.2.2. Non-Voltage-Dependent Store-Operated Ca^2+^ Entry (SOCE)

**Figure 7:**
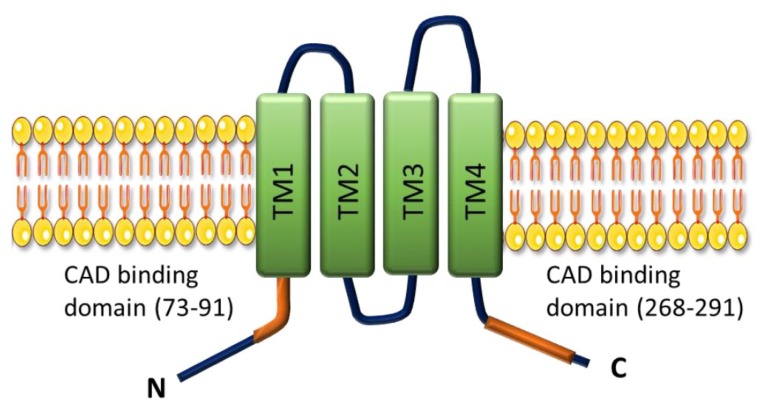
Topology and predicted domains of Orai1 channels. Each Orai1 monomer consists of four transmembrane domains (TM1–TM4) and presents CAD binding domains in the cytosolic NH2 and COOH termini.

SOCE was initially described in 1986 by Putney [92] in non-excitable cells, this discovery demonstrates that the opening of plasma membrane Ca^2+^ channels is related to the level of ER Ca^2+^ store depletion. In the last decade, two major ubiquitous SOCE components have been identified: the stromal interacting molecule-1 (STIM1) [93] and the Ca^2+^ channel Orai1 [94]. STIM1 is a single-spanned transmembrane protein that senses the ER/SR Ca^2+^ concentration predominantly located in the ER membrane and which senses the luminal Ca^2+^ concentration. Orai1 allows Ca^2+^ entry corresponding to the archetypal Ca^2+^ release-activated Ca^2+^ channels (CRAC). STIM1 has a homolog, STIM2, while the Orai family comprises three isoforms, Orai1, Orai2 and Orai3 (Figure 7).

**Figure 8:**
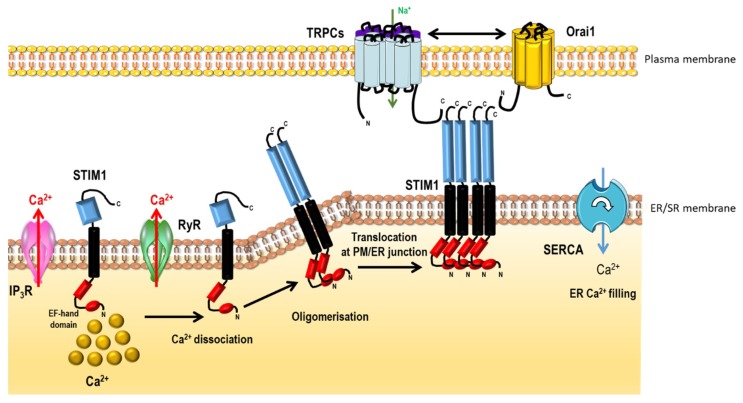
Activation mechanism of store-operated Ca^2+^ channels. The reduction in intra-luminal Ca^2+^ concentration triggers STIM1 aggregation and their interaction with Orai1 and/or TRPCs, enabling Ca^2+^ (TRPC/Orai) or cationic (TRPC) entry. TRPCs, Transient receptor potential-canonical; IP3R, inositol 1,4,5-trisphosphate receptor; RyR, Ryanodine Receptors; ER, Endoplasmic reticulum; STIM1, stromal interaction molecule; SERCA, Sarcoplasmic/endoplasmic reticulum Ca^2+^ ATPase.

After ER Ca^2+^ depletion, STIM1 oligomerizes and forms punctae and translocates close to the plasma membrane where it binds to and activates Orai1 [95] (Figure 8). In several cell types, another family of ion channels is described to co-exist and participate with SOCE, the TRPC (“canonical”) subfamily from the transient receptor potential (TRP) channel family.

**Figure 9:**
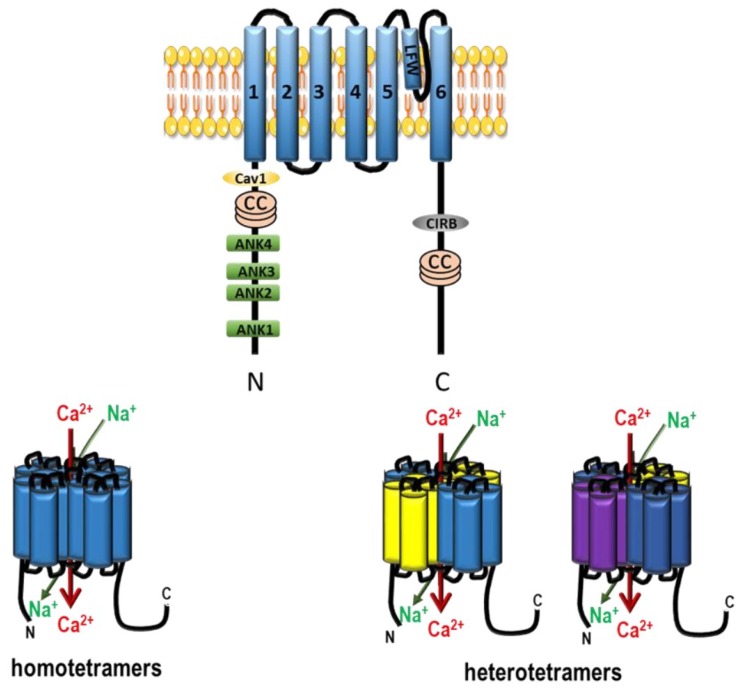
Architecture of TRPC channels. Each TRPC isoform is constituted of six transmembrane-spanning ion channels. Each TRPC subunit contains six transmembrane domains with the pore region (LFW, pore motif) between 5 and 6. Structural domains for channel assembly and protein interaction sites are located in the intracellular N and C termini. ANK1-4, ankyrin-like repeats; CC, coiled-coil domain; Ca_v_1, caveolin-1 binding domain; CIRB, calmodulin–IP3R binding site. TRPC channels need homotetramerization or heterotetramerization to form a functional channel.

The TRP channels are composed of 28 members that are divided into seven subfamilies based on sequence homology [96] (Figure 9): the TRPC (“canonical”) family, the TRPV (“vanilloid”) family, the TRPM (“melastatin”) family, the TRPP (“polycystin”) family, the TRPML (“mucolipin”) family, the TRPA (“ankyrin”) family, and the TRPN (“nompC”, no mechanoreceptor potential C) family. With the exceptions of TRPM4 and TRPM5, which are only permeable to monovalent ions, all TRP channels are cationic channels permeable to Ca^2+^.

In several cell types, STIM1 or STIM2 are able to activate Orai and TRPC channels (Figure 8).

All TRP channels contribute to the modulation of [Ca^2+^]*_i_* either directly by supporting Ca^2+^ influx through the plasma membrane, or indirectly by modulating the resting membrane potential, which controls the driving force for Ca^2+^ entry. In addition to STIM1 and Orai1, TRPC family is known to constitute an alternative and/or additional Ca^2+^ influx pathway in several cellular systems including PASMC and pulmonary ECs.

#### 2.2.3. Non-Voltage-Dependent Store-Independent Ca^2+^ Entry (SICE)

SICE are involved in the oscillatory Ca^2+^ signals during activation of the cells by low agonist concentrations (100 nM–1 μM). SICE is described to be carried by Orai1 and Orai3 proteins. Low doses of the agonist (physiological concentration) activates phospholipase A2 (PLA2) leading to the production of arachidonic acid (AA). AA activates Ca^2+^ channels constituted by a pentamer of the Orai1/Orai3 via the pool of STIM1 located in plasma membrane (20% of total STIM1) [97]. This influx is also referred to as arachidonate-regulated-Ca^2+^ entry (ARC) [98,99]. In vascular smooth muscle cells, SICE is insensitive to AA but is activated by one of its metabolites, such as the leukotriene C4. In these cells, SICE is carried by the Orai3 channel [100]. TRPCs can also be activated independently of store depletion. TRPC3, -C6, and -C7 are directly activated by DAG [101] or by phosphorylation via PKC or Src kinases [102]. Arachidonic acid and some of its metabolites are TRPC6 but not TRPC5 stimulators [103].

##### Pulmonary Arterial Smooth Muscle Cells

Among the seven TRPC isoforms (TRPC1–7) identified, all have been detected in PASMC [104,105,106]. However, the molecular expression pattern varied considerably among studies. TRPC1, -C3 and -C6 are expressed at the mRNA and protein levels in primary human PASMC [107]. The TRPC channel, and, particularly, the TRPC1, -C4 and -C6 isoforms are upregulated in BMP2 knockdown PASMC, leading to increased Ca^2+^ entry and increased human PASMC proliferation and migration [108]. Upregulation of TRPC1 expression, associated with enhancement of SOCE, is reported during proliferation of human PASMC [104].

TRPC1 and STIM1 are functionally coupled to mediate SOCE in PASMC [109]. In vascular smooth muscle cells, the silencing of caveolin 1 by a siRNA strategy induces a concomitant decrease in TRPC1 expression, leading to a reduction of Ca^2+^ entry [110]. Like in other cell types, SOCE appears clearly controlled by STIM/Orai and TRPC complex in smooth muscle cells [111].

In isolated rat aortic smooth muscle, TRPC1 and BK_Ca_ have been shown to be co-localized and co-immunoprecipitated. Functional experiments demonstrated that SOCE mediated by TRPC1 activates BK_Ca_ leading to plasma membrane hyperpolarization. This hyperpolarizing effect of TRPC1–BK_Ca_ coupling could prevent excessive contraction of vascular smooth muscle cells (VSMC) induced by contractile agonist [112]. Li and coworkers demonstrated that knockdown of STIM1 reduces SOCE and migration/invasion of human saphenous vein smooth muscle cells. In the same study, overexpression of the dominant negative form of the TRPC5 isoform strongly reduced cell proliferation, cell invasion and cell migration of human VSMC [113]. Moreover, in aorta rat smooth muscle cells, STIM1 and Orai1 mediate CRAC current and participate in the proliferation and migration of aorta rat smooth muscle cells [114].

**Figure 10:**
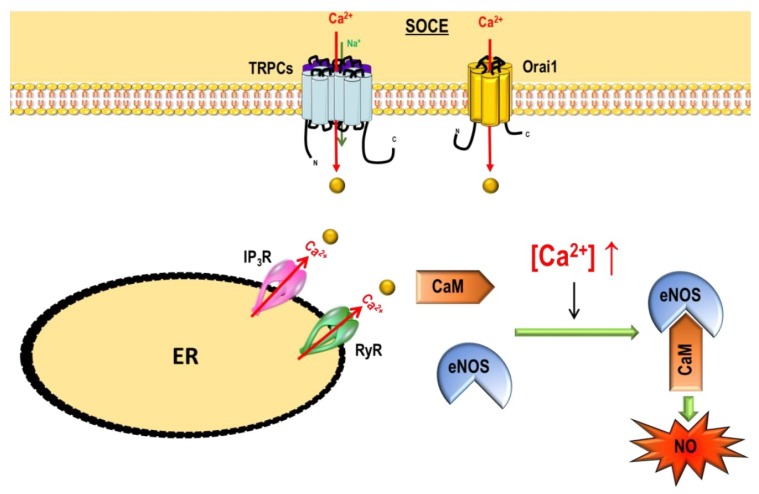
Proposed model of the regulation of eNOS activity and NO production by intracellular Ca^2+^ concentration in endothelial cells. Increased intracellular Ca^2+^ promotes interaction between calmodulin and eNOS and leads to enhanced eNOS activity and NO production under basal conditions. CaM, calmodulin; eNOS, endothelial nitric oxide synthase; NO, nitric oxide; TRPCs, Transient receptor potentialcanonical; IP3R, inositol 1,4,5-trisphosphate receptor; RyR, Ryanodine Receptors, ER: Endoplasmic reticulum.

NO is a ubiquitous intracellular signaling molecule synthesized from L-arginine by nitric-oxide synthase (NOS). Endothelial NOS (eNOS), a Ca^2+^/calmodulin-dependent enzyme, is highly regulated by [Ca^2+^]*_i_* [115]. Activation of eNOS is induced by increases in [Ca^2+^]*_i_* resulting from the activation of diverse G-protein-coupled receptors (GPCR) or from mobilization of intracellular Ca^2+^ stores (Figure 10). Interestingly, in intact endothelial cells, NO production is preferentially stimulated by SOCE [116].

##### Pulmonary Arterial Endothelial Cells

Endothelial cells exhibit a complex collection of Ca^2+^ channels, including receptor and SOCE and various Ca^2+^-permeable non-selective cation channels [28]. In ECs isolated from human lung, STIM1 is necessary and sufficient for SOCE association with TRPC1/C4 channels. Their functions are essential for mediating Ca^2^^+^ entry-dependent disruption of the endothelial barrier. Surprisingly, in lung ECs, Orai1 is not involved in SOCE [117]. In human umbilical vein ECs, the reduced expression of TRPC1, -C3, and -C4, and STIM1 impair in vitro angiogenesis, whereas siRNA against Orai1 has no effect [118].

Sporadic mutation in the caveolin-1 gene are identified in PAH patients [119] and, interestingly, caveolin-1 interacts with TRPC1 and inositol 1,4,5-trisphosphate receptor type 3 (IP_3_R3) to regulate SOCE in ECs [120]. SOCE and agonist-activated calcium entry measured in ECs from CH-exposed intrapulmonary artery are reduced compared to control and normalized with a specific PKC inhibitor (GF109203X) [121].

#### 2.2.4. Other Ca^2+^ or Cationic Channels in Pulmonary Arteries

Contrary to TRPC channels, the physiological function of other TRP channels are obscure in vascular smooth muscle. The melastatin-related TRPM (TRPM1–8) and the TRPV subfamilies (TRPV1–6) are known to participate to tumor suppression, oxidative stress/reactive oxygen species (ROS)-induced apoptosis, Mg^2+^ homeostasis, nociception, mechanosensing, osmolarity sensing, and thermos-sensing (hot and cold) in nonvascular tissues [122,123,124]. However, few studies revealed that some of these channels are also expressed in systemic vascular smooth muscles cells.

At the pulmonary arterial level, the expression of TRPM and TRPV families are examined by basic RT-PCR. The mRNA of TRPM2, -M3, -M4, -M7, and -M8 and TRPV1, -V2, -V3, and -V4 are detected in pulmonary arteries [125]. Moreover, Western blot analysis confirmed the expression of TRPM2, -M8, -V1, and -V4 proteins.

TRPM2 was found to regulate endothelial barrier permeability [126]. TRPM4 contributes to membrane depolarization and vasoconstriction associated with increased intra-luminal pressure in cerebral arteries [127]. TRPM7 is identified as a functional regulator of Mg^2+^ homeostasis in mouse and rat mesenteric and aortic smooth muscle cells [128,129]. Interestingly, the TRPV2 channel has been implicated as an osmotically sensitive cation channel in murine aorta myocytes [130].

Using Ca^2+^ imaging measurements, the TRPM8 agonist (Menthol at 300 μM) and TRPV4 agonist (4α-phorbol 12,13-didecanoate at 1 μM) evoked significant increases in [Ca^2+^]*_i_* in PASMCs. The authors suggested that these novel Ca^2+^ entry pathways may play important roles in the regulation of the pulmonary and systemic circulations [125]. TRPV4 has been demonstrated to be functionally expressed in cultured human airway smooth muscle cells, and this specific activation increases [Ca^2+^]*_i_*, which may induce smooth muscle contraction [131]. A TRPV4-like current was recorded in rat aortic smooth muscle cells and human PASMC, and the authors suggested a role for TRPV4-mediated Ca^2+^ influx in proliferation [132].

#### 2.2.5. *N*-Methyl-d-Aspartate Receptor (NMDAR)

The NMDAR, a major glutamate receptor of excitatory neuronal synapses, is an ion channel receptor also expressed in PASMC and pulmonary arterial ECs [133]. NMDARs are a class of ionotropic glutamate receptors that also includes the AMPA receptors and kainate receptors. The family of NMDARs consists of three different subunits called glutamate ionotropic receptor NMDA type subunit 1–3 (GRIN1–3) [134]. NMDAR activation leads to opening of an ion channel that is selective for cation, resulting in Na^+^, Ca^2+^ and K^+^ efflux [135]. Although most glutamate receptors are cation selective, few are permeable to Ca^2+^ ion.

At resting membrane potential, NMDARs are blocked by Mg^2+^ ions; however, if excitation by synaptic inputs causes sufficient depolarization of the neuron, the Mg^2+^ block is relieved and those NMDARs which have glutamate bound will open [135].

#### 2.2.6. Ca^2+^ Stretch Channels (Piezo1–2)

The molecular identity of non-selective stretch-activated ion channels (SACs) has long remained a mystery [28]. Recently, Piezo1 and Piezo2 channels were shown to be essential components of distinct SACs [136]. These channels are permeable to Na^+^, K^+^, Ca^2+^, and Mg^2+^, with a preference for Ca^2+^. Piezos are large transmembrane proteins conserved among various species. Piezo1 and Piezo2 are expressed in lung [137]. Defects in this gene have been associated with dehydrated hereditary stomatocytosis [138]. The pore-forming subunit of a mechanosensitive nonspecific cation channel [139,140] generates current characterized by a linear current-voltage relationship that is sensitive to ruthenium red and gadolinium.

Piezo1 is also expressed in the endothelium of developing blood vessels, and its genetic deletion profoundly alters vascular architecture [141,142,143]. Mechanosensation by Piezo1 is involved in the control of blood pressure [144]. Piezo1 is also expressed in arterial smooth muscle cells, which is involved in arterial remodeling in hypertension [145]. Regarding the crucial role of Piezo1 in systemic arterial remodeling and in endothelial shear stress, it could be interesting to study the role of Piezo channels in pulmonary circulation in context of PAH

**Figure 11:**
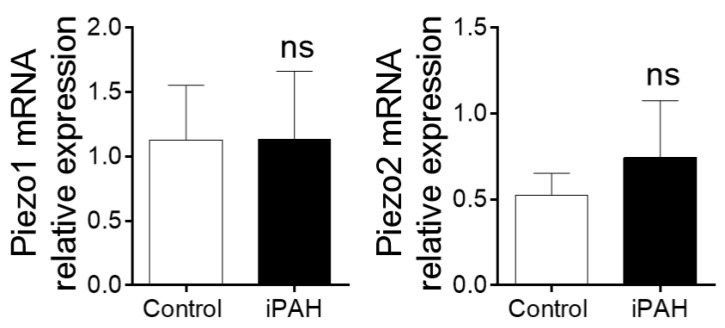
mRNA expression of Piezo1 and Pizeo 2. Unchanged expression of Piezo1 and Piezo2 mRNA expression in isolated pulmonary arteries from control (*n* = 5) and iPAH patients (*n* = 5). Gene expression was quantified using qPCR following the standard protocol for ready-to-use TaqMan gene expression assays (Life Technologies) on a StepOne Plus Real-Time PCR System (Life Technologies). Piezo1 and Piezo2 mRNA expression were normalized by β-Actin mRNA (Hs00207230_m1, Hs00926218_m1, Hs01060665_g1).

Using quantitative-PCR experiments, we revealed that Piezo1 and Piezo2 are similarly expressed in human isolated pulmonary artery from non-PAH patients and idiopathic PAH (Figure 11). Nevertheless, the function of Piezo channels in the pathogenesis of PH is currently unknown.

### 2.3. Na^+^ Channels

#### 2.3.1. Epithelial Na^+^ channels (ENaC)

Originally described in the rat colon [146], evidence has accumulated in the last decade indicating a broader expression of the ENaCs which may reveal extended physiological and pathophysiological roles. ENaC consists of at least three subunits: α, β, and γ. All three subunits are required to form a functional ENaC channel complex [147]. Administration of antagonists of ENaCs, amiloride and benzamil, stops myogenic constriction of blood vessels [148], suggesting a potential role of ENaCs in mediating vascular tone.

ENac is expressed in a variety of ECs types [149]. Although ENaC subunits are expressed in vascular smooth muscle cells and their inhibition with amiloride and benzamil reduces the myogenic response [150]. Moreover, the presence of a functional ENaC channel in PASMC has not been reported.

#### 2.3.2. Voltage-Gated Na^+^ Channels

Voltage-gated Na^+^ channels play a major role in regulating cell excitability, particularly as it pertains to the generation of action potentials in neurons, skeletal muscle, and cardiac myocytes. Nonetheless, Na^+^ currents (*I*_Na_) have been measured in visceral smooth muscles cells as well as in several vascular smooth muscles including PASMC [151].

Human PASMC expresses SCN2A to SCN11A subunits (Sodium Voltage-Gated Channel Alpha Subunit 2-11A). In human PASMC, *I*_Na_ is TTX-sensitive [151] suggesting that these channels could participate to the regulation of the resting membrane potential, excitation–contraction coupling, and cell volume. Interestingly, some studies reported that Voltage-gated Na^+^ channels are functionally expressed in human ECs (HUVEC) regulating in vitro angiogenesis and cell proliferation [152]. Traub and collaborators suggested that shear stress directly stimulates voltage-gated Na^+^ channels [153]. To our knowledge, the expression and function of voltage-gated Na^+^ channels have not been investigated in pulmonary arterial ECs yet.

### 2.4. Cl^−^ channels

The estimated equilibrium potential for Cl^−^ (*E*_Cl−_) is between −30 and −20 mV in smooth muscle cells. Physiological resting membrane potential in vascular and non-vascular smooth muscle cells ranges between approximately −60 mV and −40 mV. Cl^−^ channel activation would result in Cl^−^ efflux, leading to membrane depolarization, voltage-dependent Ca^2+^ channel activation, an elevation in [Ca^2+^]*_i_* and contraction.

PASMCs express various Cl^−^ currents, including the voltage-sensitive, volume-regulated and Ca^2+^-activated Cl^−^ currents and Cystic Fibrosis Transmembrane Conductance Regulator (CFTR).

Ca^2+^-activated Cl^−^ (ClCa) currents play a role in the myogenic response in cerebral arteries [154]. Over the past two decades, unrelated Cl^−^ channel genes families have been postulated to form or share the molecular basis of ClCa:

(i) CLC family, the long human isoform variant of CLC-3, a member of the voltage-gated Cl^−^ channel superfamily of Cl^−^

(ii) Bestrophin (BEST) gene; mutations in this gene are responsible for juvenile-onset vitelliform macular dystrophy

(iii) TMEM16 or anoctamin gene family 

Concerning CLC-3, Liang and colleagues demonstrated that a siRNA strategy against CLC-3 increased proliferation of human PASMC [155]. Very little is known regarding the functional role of bestrophins in VSMC, however Best1–4 are described to be expressed in several smooth muscle cells including PASMCs [156].

#### 2.4.1. TMEM16A Family

In 2008, three independent groups of investigators showed that two members of the *TMEM16* gene family, *TMEM16A* or *anoctamin-1* (*ANO1*) and *TMEM16B* or *anoctamin-2* (*ANO2*), encoded for ClCa [157,158,159]. The role of anoctamins in determining pulmonary arterial tone is still under evaluation. *ano2* knockout mice develop and breed normally, exhibiting an apparently normal phenotype [160]. However, *ano1* knockout mice die prematurely after birth due to malformation of their airways during embryonic development [161].

In larger blood vessels (e.g., aorta and carotid artery), an inducible VSMC-specific knockout of *tmem16A* in mice had reduced contractility to angiotensin II and U46619 (thromboxane analog) compared with their wild type counterparts without any effect on mesenteric small artery contractility [162]. In the systemic circulation, TMEM16A endothelial-specific knockout in transgenic mice showed the specific involvement of TMEM16A in regulating endothelial function and blood pressure via vascular oxidative stress [163]. The knockout of endothelial-specific TMEM16A significantly lowered the systemic blood pressure and ameliorated endothelial dysfunction in angiotensin II-induced hypertension, whereas the overexpression of endothelial-specific TMEM16A resulted in the opposite effects. *TMEM16A* gene is expressed in rat pulmonary arteries [164].

Indubitably, Ca^2+^ release from internal Ca^2+^ stores represents an important source of Ca^2+^ for ClCa activation. Evidence from several laboratories suggested that ClCa could be functionally coupled to plasma membrane Ca^2+^ channels [165]. Interestingly, in human epithelial cells, TRPC6 and ClCa are functionally coupled [166], and TRPC6 contributes to PH pathogenesis (see Section 3). Moreover, in salivary gland cells, TRPC1 is suggested to be essential for the activation of ClCa [167]. Orai1-mediated SOCE is necessary for agonist-induced chloride secretion and activation of the ClCa, ANO1 [168]. Another conceivable activation mechanism of ClCa could be through TRPV4-mediated Ca^2+^ influx which physically interact with this anion channel. Obviously, in excitable cells including PASMC, voltage-gated Ca^2+^ channel Ca_v_1.2 represents an important source of Ca^2+^ stimulating ClCa. Indeed, evidence of a coupling between Ca_v_1.2 and ClCa is already reported in smooth muscle cells isolated from different rodents’ arteries [165] including PA from rabbit and rat [169].

#### 2.4.2. Cystic Fibrosis Transmembrane Conductance Regulator (CFTR)

The CFTR is a cyclic adenosine monophosphate (cAMP)-dependent chloride channel expressed at the apical membrane of epithelial cells. CFTR is mainly studied in epithelial cells, however some reports revealed a CFTR expression in non-epithelial tissues as well as cardiac muscle cells, endothelial cells, and smooth muscle cells [170]. Robert and colleagues demonstrated that CFTR channels are expressed in pulmonary arteries and contribute to endothelium-independent relaxation in rat pulmonary arteries [170]. Moreover, Totani et al. demonstrated that CFTR is functionally expressed in human PAEC [171].

## 3. Ion Channels in PAH Pathophysiology

Pulmonary vascular cells from PAH are characterized by an important remodeling of ion channels, at both the gene/protein expression and function. These changing are summarized in Table 2.

### 3.1. K^+^ Channels

#### 3.1.1. Voltage-Gated K^+^ Channels in PAH

Since work done by Yuan et al. in 1998, a role for K^+^ channels in PAH pathobiology has been suggested [44]. These results were further supported by the identification of a SNP in the *KCNA5* gene (encoding K_v_1.5) in systemic sclerosis-associated PAH, which alters the function and/or the expression of K_v_1.5 channels [16]. The decrease in K_v_1.5 expression is associated with plasma membrane depolarization and an increase in [Ca^2+^]*_i_* [44]. Mutations in *BMPR2* are responsible for heritable PAH [172]. A decrease in mRNA expression for K_v_1.1, K_v_1.5, and K_v_4.3 is found in lungs of patients carrying a *BMPR2* mutation. In human PASMC treated with recombinant BMP2, the K_v_1.5 protein and macroscopic K_V_ current density is strongly increased [173].

In rat PASMC, the overexpression of K_v_1.5 channels increases K^+^ currents and leads to plasma membrane hyperpolarization. Interestingly, acute hypoxia reduces K_v_ currents in K_v_1.5 overexpressing PASMC, demonstrating that the K_v_1.5 isoform is a hypoxia-sensitive K_v_ channel in PASMC [174]. K_v_1.5 contributes to the development of exaggerated chronic hypoxia PH in intrauterine growth retardation rat models [175].

In cultured rat PASMC, hypoxia exposure reduces expression of K_v_1.1, -1.2, -1.5, -2.1, -4.3, and -9.3 subunits [21]. Sedivy et al. demonstrated a role of K_v_7 channels in responses of the pulmonary circulation to hypoxia [176] and demonstrated that flupirtine (a K_v_7 activator) could reverse experimental PH induced by CH exposure in mice [177]. Together, these results suggest that K_v_7 activators could be beneficial in PH. Wang J demonstrated that the effects of CH on K_v_ channel expression are specific to rat pulmonary arteries compared to rat aorta PASMC [178].

Moreover, K_v_1.2 channels are expressed in small rat pulmonary arteries and produce a significant current in native rat PASMC. The application of antibody against the K_v_1.2 isoform reduced K^+^ current in normoxic conditions and had no effect on reduced-hypoxic K^+^ current, implicating this channel in hypoxic pulmonary vasoconstriction [179].

#### 3.1.2. Ca^2+^-Activated K^+^ Channels in PAH

Hypoxia enhances cAMP-mediated BK_Ca_ channel activation in adult murine PASMC, suggesting that modulation of this pathway could modulate adult pulmonary vascular tone after perinatal hypoxia [180]. An upregulation of the BK_Ca_ channels at the mRNA level has been shown in remodeled pulmonary arteries of IPAH patients. In this line, DHA (docosahexaenoic acid) hyperpolarized the membrane potential of human PASMCs from IPAH patients, which was depolarized at baseline. Moreover, in a CH PH model, activation of BK_Ca_ channels with acute DHA reduced the elevated right ventricular systolic pressure, demonstrating that activation of BK_Ca_ is a potent modulator of pulmonary vascular tone [181].

In pulmonary arteries isolated from chronically hypoxic rats, K_Ca_3.1 channel is downregulated and associated to the impairment of pulmonary arteries relaxation [64].

#### 3.1.3. K_2P_ Channels in PAH

In 2013, six different mutations in *KCNK3* gene were identified in PAH patients, increasing the interest in ionic channels in the development of this pathology [11]. More recently, two additional mutations were reported in a Spanish cohort [13], one in a Japanese cohort [14,15] and two in a British cohort [14].

To date, 10 different mutations in *KCNK3* gene have been identified in 15 different PAH patients (nine women and six men) (Table 3). Only one patient with a homozygous *KCNK3* mutations has been reported, which was associated with an aggressive form of PAH and an early development of the disease. *KCNK3* mutations have been identified in patients aged 8–52 years [11,13,14,15]. As presented in Table 3, and similar to *BMPR2* carriers with PAH as described by Evans et al. [182], *KCNK3* mutations carriers patients are younger than idiopathic patients at diagnosis (mean age 29 ±11.22 vs. 42 ± 17.8 years) and have a higher mean pulmonary artery pressure (76 ± 17.95 vs. 56.4 ± 15.3 mmHg) [14].

Electrophysiological experiments revealed a loss of function of all identified *KCNK3* mutants [11,12]. In their study, Ma and colleagues also demonstrated that this loss of function of some *KCNK3* mutants (*T8K*, *E182K* and no *G203D*) can be reactivated with the use of a phospholipase A2 inhibitor (ONO-RS-082), indicating that this is not a case of a complete loss of function. As recently reviewed by Olschewski et al., in PAH pathobiology, ET-1 is shown to inhibit KCNK3/TASK-1 in human PASMC via Rho kinase phosphorylation [183]. It is well established that ET-1 is strongly increased in plasma from PAH patients, contributing to the development of PAH. In explanted culture of human PASMC, functional KCNK3/TASK-1 expression appears downregulated in hypoxia conditions as compared to normoxia, and this phenomenon is dependent of Src tyrosine kinase activity [184].

We recently demonstrated that KCNK3/TASK-1 expression and function are reduced in human patients with PAH (idiopathic PAH and heritable PAH carrying a *BMPR2* mutation) and in monocrotaline-induced PH in rats. We confirmed that KCNK3/TASK-1 contributes to the resting membrane potential of PASMC, which is already shown in human PASMC [68]. We also demonstrated that pharmacological inhibition of KCNK3/TASK-1 with A293 (a specific KCNK3/TASK-1 inhibitor provided by Sanofi) highlighted that KCNK3/TASK-1 channels modulate pulmonary arterial tone. Long-term in vivo pharmacological inhibition of KCNK3/TASK-1 in rats induced distal pulmonary vessel neomuscularization and early hemodynamic signs of PH, which are related to exaggerated proliferation of pulmonary artery ECs, PASMC, adventitial fibroblasts, and pulmonary and systemic inflammation. Lastly, in vivo pharmacological activation of KCNK3/TASK-1 with ONO-RS-082 significantly prevented the development of PH in monocrotaline-exposed rats [76]. More recently, ONO-RS-082 (10 µmol/L) was confirmed to activate both wild-type and mutant *KCNK3* channels, causing cell hyperpolarization. Bohnen et al. demonstrated that KCNK9 minimizes the impact of *KCNK3* mutations when the two channel subunits co-assemble. These data suggest that *KCNK9* gene transfer in the pulmonary vasculature could be a beneficial approach to alleviate PH (KCNK9 being not expressed in lung) [12].

**Figure 12:**
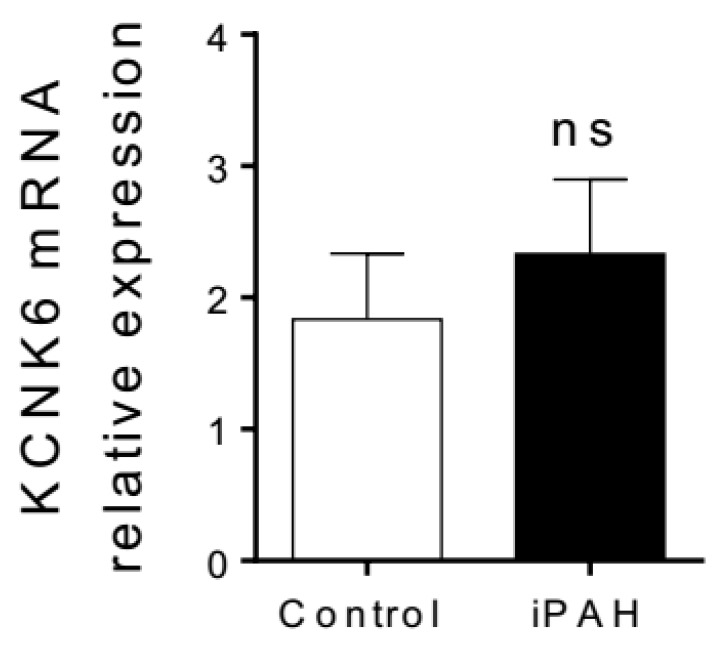
mRNA expression of KCNK6. Unchanged expression of KCNK6 mRNA in isolated pulmonary arteries from control (*n* = 5) and iPAH patients (*n* = 5). Gene expression was quantified using qPCR following the standard protocol for ready-to-use TaqMan gene expression assays (Life Technologies) on a StepOne Plus Real-Time PCR System (Life Technologies). KCNK6 mRNA expression was normalized by β-Actin mRNA (Hs00559239_g1 and Hs01060665_g1).

Compelling evidence suggests that the mouse is not a suitable model to study the role of KCNK3/TASk-1 in the pulmonary vasculature since KCNK3/TASk-1 has been described as non-functional in murine PASMC [75,185]. In mice, kcnk3 seem to be replaced by the *kcnk6* isoform. *kcnk6* deficient mice develop spontaneous PH between 8 and 20 weeks of age through a mechanism involving Rho-kinase [186]. The authors proposed that downregulation of KCNK6 in the pulmonary vasculature could be an underlying mechanism in PAH. To investigate the expression of KCNK6, we performed RT-qPCR experiments on isolated pulmonary arteries from idiopathic PAH and control patients. As shown in Figure 12, mRNA expression of KCNK6 is unchanged in pulmonary arteries from iPAH patients (Figure 12) contrary to KCNK3 expression [76].

### 3.2. Ca^2+^ Channels

#### 3.2.1. Voltage-Gated Ca^2+^ Channels in PAH

VGCC have already been documented in a therapeutic target context of neuropathic and neuropsychiatric diseases [84]. In experimental PH induced by CH, PASMC vasoconstriction and proliferation are associated with an increase in [Ca^2+^]*_i_* mainly due to upregulation of L-type channels and L-type channel antagonists prevent hypoxic pulmonary vasoconstriction [187]. The vasoconstrictor ET-1 is known to increase [Ca^2+^]*_i_* through L-type channel activity in normoxic conditions [188]. In CH PH-rats, ET-1 is markedly increased in PASMC during PH progression [189] and is associated with an increase in [Ca^2+^]*_i_* independently to L-type channel activity [188]. However, a more recent study demonstrated that ET-1 signaling is altered in PASMC isolated from CH rats, inducing activation of L-type channels through activation of PKC, Rho kinase and tyrosine kinase [190]. Of interest, an inhibition of L-type channels by CCB medications, such as nifedipine, can be used as long-term therapy for PAH patients responding positively to an acute vasodilator test [191]. Alternatively, the microRNA-328, which has multiple gene targets, inhibited Ca_v_1.2 expression, protects from pulmonary artery vasoconstriction and remodeling in hypoxic exposure, providing a novel therapeutic insight for PAH treatment [192].

Besides L-type channels activities, T-type channel, *Ca_v_3.2*, has been shown to be enhanced in human PASMC [81], in PASMC from hypoxic-induced PH mouse model [193] and from CH PH-rats exposure. In PH induced by monocrotaline exposure, both L- and T-type channel activities are responsible for electrical remodeling that alters excitation–contraction coupling [194].

Several mediators of PAH pathobiology (Angiotensin II, ET-1, and Serotonin [195]) have been shown to upregulate T-type channel expression/activity [80,196,197,198]. In mice with carotid arterial injury, *I*_CaT_ is required for neointimal formation. Then, according to the expected T-type channel function in the cell process, altered T-type channels activity might account for aberrant cell processes such as hyper-proliferation and resistance to apoptosis. Recent studies assessing the role of *I*_CaT_ in PAH progression using experimental animal models, or PASMC from PAH patients, lead to the emergence of T-type channels as potential therapeutic targets in PH.

Hypoxia and monocrotaline-induced PH are associated with a depolarization of PASMC mainly explained by a decrease in K_v_1.5 and KCNK3 [21,76]. Since T-type channels Ca_v_3.1 and Ca_v_3.2 are low-voltage activated Ca^2+^ channels that open during membrane depolarization, the decrease in K_v_1.5 and KCNK3 channels activities should be associated with an elevation in cytosolic Ca^2+^ concentration through *I*_CaT_.

In CH PH-rats, PH is prevented by the use of in vivo T-type channel blockade [90]. The deletion of the gene encoding *Ca_v_3.1* in transgenic mice protects against the development of CH PH. Indeed, in contrast to wild-type mice, mice lacking the *Ca_v_3.1* gene do not develop pulmonary arterial wall remodeling or right ventricle hypertrophy when exposed to CH [90].

We recently studied T-type-dependent Ca^2+^ signaling in PASMC from patients with idiopathic PH (iPAH) [82]). In these experiments, we showed that T-type signaling is disrupted towards PP2A activation and readdressed to Akt1 activation. A critical impact of this impaired T-type signaling is observed on proliferation, survival and apoptosis resistance of PASMC [82]. Therefore, both increased PP2A activity and decreased kinase activity induced by T-type signaling might have beneficial effects on the onset of PAH.

#### 3.2.2. SOCE in PAH

The protein expression level of STIM2 is significantly increased in idiopathic PAH-PASMC compared to control PASMC, whereas STIM1 protein expression is not significantly modified. A siRNA strategy directed against STIM2 induces the reduction of SOCE amplitude and, consequently, decreased iPAH-PASMC proliferation. Conversely, STIM2 overexpression in control PASMC enhanced SOCE and PASMC proliferation [199]. Following this work, Fernandez and collaborators demonstrated that STIM2 and Orai2 are upregulated during the phenotypic transition of PASMC from a contractile state to a proliferative state [200]. STIM2 and Orai2 upregulation in this phenotypic transition enhance SOCE in PASMC and consequently PASMC proliferation [200]. Recently, nicotinamide phosphoribosyltransferase (NAMPT), a cytozyme, has been described as regulating intracellular nicotinamide adenine dinucleotide levels, the cellular redox state, cell proliferation and apoptosis level [201]. In addition, the recombinant NAMPT stimulates human PASMC proliferation via an enhancement of SOCE by upregulation of Orai2 and STIM2 expression [201].

SOCE appears significantly increased in rat distal PASMC compared to rat proximal PASMC. SOCE is not affected by hypoxia in proximal PASMC while Ca^2+^ is increased during hypoxia in distal rat PASMC. These results are correlated with an increase in the expression of TRPC1 and STIM1 in rat distal PASMC [202]. Very recently, Rode and colleagues discussed the interest in targeting Orai1 in the treatment of PH [203].

#### 3.2.3. TRPC Channels in PAH 

Yu and coworkers identified a unique genetic variant in the promoter of the gene *TRPC6* in few iPAH patients. This mutation may predispose to an increased risk of developing iPAH [18]. Interestingly, mRNA and protein expression of TRPC6 are significantly increased in lung tissues and PASMCs isolated from iPAH patients compared to normal subjects. Moreover, the silencing of TRPC6 downregulates the hyper-proliferative phenotype of PASMC from iPAH patients [204]. In the study by Zhang and collaborators, the authors described that SOCE is significantly increased in cultured human PASMC from iPAH patients via an increase in the expression of TRPC3 isoform [205].

In human PASMC, Tang Y and coworkers suggested that TRPC6 activation occurs subsequent to an elevation in cellular AMPK during hypoxia and acts to ensure sufficient membrane depolarization for the activation of a greater Ca^2+^ conductance via VGCC [206].

Using a specific E3-targeted antibody to TRPC1, Kumar and colleagues showed a strong reduction of neointimal growth in human veins, as well as Ca^2+^ entry and proliferation of smooth muscle cells in culture. This study suggested that an upregulation of TRPC1 activity may play a key role in occlusive vascular disease and that TRPC1 inhibitors have potential as protective agents against human vascular failure [207].

In CH PH mice, specific *trpc1* and *trpc6* gene deletion suppresses PH compared with wild-type, associated with decreased pulmonary microvessel remodeling, but capillary rarefaction is unchanged. The presence of TRPC1 and TRPC6 is essential for the full development of hypoxic PH in the mouse model. [208].

In another study, only the mRNA level of TRPC1 appears to be up-regulated after 72 h exposure to 1% O_2_ in isolated murine PASMC. This hypoxic treatment induces proliferation of murine PASMC, which is attenuated by a siRNA strategy against TRPC1 channels. In this study, *trpc1*^−/−^ mice do not develop PH in response to CH and have a similar degree of right ventricular hypertrophy compared to wild-type mice. Nevertheless, *trpc1*^−/−^ mice have less vascular muscularization [209].

After induction of PAH in *Trpc4*-knockout rats (*Trpc4*-KO) with a Sugen 5416 injection, followed by three weeks hypoxia exposure, histological grading in *Trpc4* inactivated rats revealed a reduction in the density of severely occluded small pulmonary arteries and in the number of plexiform lesions. To summarize, this unique *Trpc4*-KO rat model provided a survival benefit in severe PAH, associated with a decrease in the magnitude of occlusive remodeling [210]. Using *Trpc4*-KO rats, Francis and collaborators demonstrated that TRPC4 provides a Ca^2+^ source associated with endothelial dysfunction in the pathophysiology of PAH [211].

BMP4 application is also demonstrated to increase TRPC1, -C4 and -C6 expression, SOCE and basal Ca^2+^ concentration in rat distal PASMC cell contributing to pulmonary vascular remodeling in PAH. An increase in the expression of TRPC6 channel and Na^+^/Ca^2+^ exchanger (NCX1) is observed in freshly dissociated PASMC from Milan Hypertensive rats (MHS), which contribute to abnormal Ca^2+^ homeostasis. The authors suggested that the increase in Ca^2+^ signaling due to increased TRPC6 and NCX1 expression participates in vasoconstriction and elevates blood pressure in MHS rats [212].

TRPC1, -C3 and -C6 appear to be expressed at the mRNA and protein levels in primary human or rat PASMC. TRPC1 and TRPC6 expression are strongly increased in CH rat. By a siRNA approach, the authors demonstrated that the abnormal SOCE amplitude in hypoxic rat is reduced by siTRPC1 treatment. Moreover, the exaggerated OAG-mediated (Oleyl-Acetyl-Glycerol a DiAcylGlycerol analog) Ca2^+^ entry in hypoxic PASMC is reduced in siTRPC6 conditions. Targeting these channels may provide future treatments in hypoxia-related PH [107].

#### 3.2.4. Other TRP Channels in PAH Pathophysiology

Recently, another member of the TRP superfamily, TRPV4, has been demonstrated to contribute to 5-HT-mediated pulmonary vasoconstriction and could be an important player for the development of CH PH, thus contributing to PASMC migration and pulmonary vascular remodeling. Thus, strategies to inhibit or down-regulate TRP channels in PAH should induce therapeutic benefit by limiting pulmonary vascular remodeling.

Data obtained in *trpv4*-KO mice demonstrated that TRPV4 contributes to 5-HT-dependent pharmaco-mechanical coupling and plays a major role in the enhanced pulmonary vasoreactivity to 5-HT in CH PH. The genetic deletion of *trpv4* suppresses the development of CH PH [213].

Specific pharmacological activation of TRPV1 and TRPV4 channels significantly increased the migration of rat PASMC cells via an increase of cytosolic Ca^2+^ concentration and independently of increased cell proliferation. Specific TRPV1 or TRPV4 blockers in cultivated rat PASMC reduce migration in the development of PH [214]. The same research group showed an increased expression and current of TRPV4 isoform in cultivated PASMC of chronically hypoxic rat compared to normoxic rat [215].

Overexpression of TRPV1 channels is seen in cultured PASMC from iPAH patients compared to control patients. Associated with TRPV1 overexpression, Ca^2+^ signaling induced by capsaicin (an active component of chili pepper and TRPV1 channel activator) is enhanced in PASMC from iPAH patients contributing to over-phosphorylation of CREB in PAH. Conversely, pharmacological inhibition (with TRPV1 antagonist) or siRNA against TRPV1 attenuates aberrant iPAH PASMC proliferation [216].

#### 3.2.5. TRPM

Interestingly, three single nucleotide polymorphisms (SNPs) in the *TRPM8* gene (rs9789675, rs9789398, and rs1004478) are significantly associated with the risk of PH in Han Chinese patients with chronic obstructive pulmonary disease (COPD) [217].

#### 3.2.6. Acid-Sensing Ion Channels (ASIC)

ASICs are voltage-independent amiloride-sensitive and proton-gated cation channels, and their activity has been linked to a variety of physiological and pathological functions in the central and peripheral nervous system. The ASIC family of channel is constituted by five genes (ACN1–ACN5) encoding at least seven subunits (ASIC1a, -1b, -2a, -2b, -3, -4 and -5) [218]. The subunit composition of ASIC mainly determines the pH sensitivity and gating characteristics of the channel. However, Acid sensing ion channel 1 (ASIC1) is expressed in a variety of tissues including PASMC, whose contribute to enhanced Ca^2+^ entry mediating vasoconstriction, vascular remodeling, and right ventricular hypertrophy associated with hypoxic PH [218,219]. ASIC channels are mainly permeable to Na^+^ but also conduct Ca^2+^ [219]. Consequently, the influx of Na^+^ and Ca^2+^ contribute to membrane depolarization. Moreover, ASIC1 is also demonstrated to participate in SOCE and to contribute to aberrant SOCE in PASMC from CH mice [220,221] and to mediate activation of the NFATc3 transcription factor [222]. In conclusion, ASIC1 channel represents another attractive channel for PAH treatment.

#### 3.2.7. Ca^2+^-Sensing Receptor (CaSR)

CaSR is a G-protein coupled receptor (GPCR). The CaSR functions as a dimer in which the venus-flytrap-like domains of each monomer interact. Ca^2+^ binds in the cleft of the Venus-flytrap-like domain and causes a conformational change in CaSR, inducing cell signaling events [223]. Recently, CaSR was shown to be upregulated in PASMC and lung tissues from iPAH patients [224,225]. This work indicates that CaSR is functionally coupled with TRPC channels to mediate Ca^2+^ influx and increased [Ca^2+^]*_i_*, leading to exacerbated proliferation of iPAH-PASMC [226]. This demonstrates the pathogenic role of CaSR in PAH genesis.

#### 3.2.8. Other Non-Voltage Gated Ca^2+^ Channels

In all cell types, intracellular Ca^2+^ homeostasis is also regulated by the activity of Sarcoplasmic reticulum Ca^2+^ ATPase (SERCA). In lung samples from PAH patients, in cultured human PASMC isolated from iPAH patients and from the monocrotaline (MCT) rat model, the expression level of the SERCA2a isoform is strongly reduced [227]. Remarkably, Hadri and coworkers restored SERCA2a expression in rat MCT by intratracheal delivery of aerosolized adeno-associated virus serotype 1 (AAV1) carrying the human SERCA2a [227]. In vitro, this gene transfer decreases proliferation and migration of hPASMC, and in MCT-PAH rats in vivo, the AAV1.SERCA2a treatment strongly reduced pulmonary arterial pressure, vascular remodeling, right ventricular hypertrophy and fibrosis compared to MCT-PAH rats [227]. These genes transfer approaches may be a fascinating approach to moderate the development of PAH.

Recently, we reported a novel association of PAH with another channelopathy due to mutation in *ATP1A2* gene, known to predispose to familial hemiplegic migraine [228]. This gene encodes the α2-subunit of the Na^+^/K^+^-ATPase, reinforcing the interest of ion channels in the pathophysiology of PAH.

In 2018, rare variants of additional putative genes *ATP13A3* were found [10]: *ATP13A3* code for a member of the P-type ATPase family of proteins that transport a variety of cations across membranes.

#### 3.2.9. NMDAR in PAH

Recently, NMDAR expression was demonstrated in human and rodent pulmonary vascular cells [133], which contributes to vascular cell proliferation and angiogenesis. In PAH, glutamate (a NMDAR agonist) is accumulated in pulmonary arteries from PAH patients. Moreover, NMDAR antagonists and *nmdar*^−/−^ mice reduce the development of PH, demonstrating that a dysregulated glutamate/NMDAR axis in pulmonary arteries leads to PAH. Thus, vascular NMDAR is a potential treatment target for PAH [133].

### 3.3. Na^+^ Channels

In the context of PAH where rennin-angiotensin-aldosterone system (RAAS) is dysregulated [195], ENaC is an end-effector in the RAAS contributing to reabsorption of Na^+^ in renal cortical collecting ducts [141].

Plastohyn et al. revealed that Na^+^ voltage-gated channel subunit 1B (SCN1B) is upregulated in pulmonary vascular tissues from idiopathic PAH patients, which could contribute to the abnormal vasoconstriction and excessive pulmonary vascular remodeling observed in PAH patients [151].

### 3.4. Cl^−^ Channels

#### 3.4.1. CFTR

Tabeling and colleagues recently demonstrated that mice lacking *cftr* in the lungs are partially protected against CH-induced PH and pulmonary arterial remodeling, possibly via CFTR/TRPC6 protein complex formation [229]. In human epithelial cells, CFTR and TRPC6 are also functionally coupled [230]. They identified the critical role of CFTR as a chaperone for the trafficking of transmembrane ion channels, a mechanism which may equally pertain to other hypoxia-related lung diseases commonly associated with PH, such as COPD or lung fibrosis. In line with the notion that CFTR interacts with other ion channels, the functional complex of TASK-1/CFTR for regulation of chloride secretion in shark rectal gland has been demonstrated [231].

#### 3.4.2. Ca^2+^ Activated Cl^−^ Channels 

Augmented ROCE and SOCE and in some cases increased expression of several members of the TRPC gene family as well as upregulation of VGCC activity are reported in PH. They are sources of increased intracellular Ca^2+^ activating ClCa channels that lead to increased SMC excitability by promoting membrane depolarization, further activation of voltage-dependent Ca^2+^ channels, increased Ca^2+^ influx, and vasomotor tone [165]. In the monocrotaline (MCT)-induced PH rat model, whole cell patch experiments revealed an increase in niflumic acid (NFA)-sensitive Ca^2+^-activated Cl^−^ current density in PASMCs from large conduit and small intralobar pulmonary arteries of MCT-treated rats [232]. Quantitative RT-PCR and Western blot analysis showed that the alterations in current density are accompanied by parallel changes in the expression of TMEM16A. In the CH PH rat model, enhanced Ca^2+^-activated Cl^−^ current is detected upon caffeine-induced Ca^2+^ release or by depolarization at a constant high intracellular [Ca^2+^] (500 or 750 nm) and is associated with increased TMEM16A mRNA and protein expression [233]. In endothelium-denuded pulmonary arteries of CH rats, the enhanced contractile response is prevented by T16A(inh)-A0a (a TMEM16A inhibibitor) and by the Ca^2+^-activated Cl^−^ channels niflumic acid but also by the L-type Ca^2+^ channel antagonist nifedipine, supporting the relevance of the Ca^2+^-activated Cl^−^ channels in vasomotor tone in the pulmonary circulation under disease conditions.

## 4. Targeting Ion Channels and Transporters in PAH

### 4.1. Direct Pharmacological Action on Ion Channels in PAH

PAH is mainly characterized by hyperproliferative and vasoconstrictive PASMC, involving several abnormalities in ion channel function and expression. Drugs acting directly on ion channels could be attractive for reducing aberrant pulmonary vascular cell proliferation and vasoconstriction.

#### 4.1.1. Voltage Gated Ca^2+^ Channels

The only ion channels currently targeted by drug therapy in PAH are the L-type VGCC. Inhibition of L-type Ca^2+^ influx by nifedipine, diltiazem or amlodipine may reduce excessive pulmonary arterial vasoconstriction, however, those molecules also dilate non-pulmonary arteries.

Regrettably, VGCC blockers are only effective in a small subgroup of iPAH. These responders are defined by a fall of 10–40 mmHg in mPAP during inhalation of NO [191].

#### 4.1.2. K_v_1.5 Gene Therapy

The functions of the K_v_1.5 gene in carcinoma progression have also provoked a flurry of research in the hope of developing adjunctive or combined treatment of K_v_1.5 modulators to enhance curative effects of classic chemotherapeutic agents.

Another interesting strategy could be gene therapy. Indeed, in vivo gene transfer of the K_v_1.5 potassium channel reduces PH and restores hypoxic pulmonary vasoconstriction in chronically hypoxic rats [234].

#### 4.1.3. K_ATP_ Channels

In a preventive strategy in MCT exposed rats, daily treatment with iptakalim, a new adenosine triphosphate (ATP)-sensitive potassium channel (K_ATP_) opener, attenuated PH induced by MCT [235]. Linking to these results, iptakalim inhibited endothelin-1-induced proliferation of human PASMC through the activation of K(_ATP_) channel, indicating that iptakalim may be a promising candidate for the treatment of PAH [236].

### 4.2. Indirect Pharmacological Action on Ion Channels in PAH

#### 4.2.1. KCNK3/TASK-1

In 2007, a patent explored the molecules associated with phosphorylation of the human KCNK3 channel. Several molecules able to modulate human KCNK3 have been found, including the phospholipase A2 inhibitor ONO-RS-082. In the work identifying KCNK3 mutations in PAH, Ma and colleagues demonstrated that ONO-RS-082 is able to re-activate some KCNK3 mutants, suggesting that this molecule could be a beneficial therapeutic approach to target KCNK3 in PAH [11]. In vitro, using the whole cell patch-clamp technique, we confirmed that ONO-RS-082 (10 μmol/L) improved endogenous rat KCNK3 current. In accordance to the loss of KCNK3 expression in experimental PH (MCT model), the ONO-RS-082 curative treatment is inefficient at reduced PH symptoms in MCT-PH. However, a preventive treatment with ONO-RS-082 at 50 mg/kg/day improved experimental PH symptoms (MCT model) at hemodynamics levels, RV hypertrophy, and pulmonary vascular remodeling [76]. These results highlighted that this strategy might be of interest in patients with PAH who retain a residual level of KCNK3 expression, possibly within the frame of personalized therapy. The major limitation is that ONO-RS-082 compound is also a phospholipase A2 inhibitor, which could have serious side effects. Identification or development of more specific KCNK3 activators are needed to envision this pharmacological approach in humans.

The powerful vasodilator prostacyclin analogs iloprost and treprostinil activate KCNK3/TASK-1 current via a protein kinase (PK) A-dependent pathway, representing an important mechanism of the vasorelaxing properties of prostanoids [65].

Finally, we showed that endothelin-1 (ET-1), a potent vasoconstrictor for vascular remodeling, inhibit KCNK3/TASK-1 current via a protein kinase (PK) C-dependent pathway in human PASMCs [237]. Thus, the beneficial effect of endothelin receptor antagonists in PH can be partially explained by the abolishment of the specific effect of ET-1 via specific G-protein-coupled receptors on KCNK3/TASK-1.

Beside the beneficial drug effects on KCNK3/TASK-1 in PH, there are also several medicament-induced pathways leading to the development of PH. In human primary PASMC, Src tyrosine kinases (SrcTKs) are co-localized with the KCNK3/TASK-1 channel. The ability of SrcTK to modulate the channel function under resting condition as well as under hypoxia provides evidence for the importance of SrcTK in regulating the membrane depolarization and intracellular calcium rise. Consequently, it is very likely that SrcTK as the essential regulator for K^+^ channel including KCNK3/TASK-1 activity plays a pivotal role for the development of potent inhibitor of SrcTK dasatinib-induced PAH [184,238,239].

#### 4.2.2. K_ATP_ Channels

Levosimendan, a drug recently introduced into clinical practice for treatment of right ventricular decompensation, significantly attenuated the increased pulmonary vascular medial wall thickness after monocrotaline challenge in rats [240]. Accordingly, levosimedan diminished proliferation of PASMCs in vitro and this effect is attenuated by glibenclamide suggesting that the effect is K_ATP_-dependent.

#### 4.2.3. Ca^2+^-Activated K Channels (K_Ca_)

The vasodilatory pathway activated by NO targets several ion channels. Inhibition of cGMP (cyclic guanosine monophosphate) breakdown or stimulators and activators of sGC are effective strategies in PH. They increase and prolong the vasodilatory effect of cGMP leading to vasodilation and improved clinical outcome by mainly activating K_Ca_ in PASMCs [183].

#### 4.2.4. TRPC1 and TRPC6

The PDE-5 inhibitor sildenafil has been shown to reverse the PH-induced upregulation of TRPC1, TRPC6 as well as SOCE, and to decrease Ca^2+^ responses to various vasoconstrictors molecules [241].

### 4.3. Untested Pharmacological Compounds in PAH

As summarized in Table 2, remodeling of ion channels in PAH involves loss or over expression of several ion channels. Direct pharmacological activation of ion channels appears complex when channels expression is lost (mostly for K^+^ channels). However, channel overexpression or over activity could be targeted by existing pharmacological tools.

#### 4.3.1. K_v_ Channels

For K_v_ channels, and due to redundancy of K_v_ channel (because they belong to a large multigenic family), pharmacological activation of unaltered K_v_ isoforms could be an interesting way of action. In CH mice, Flupirtine application (30 mg·kg^−1^·day^−1^), reverses experimental PH [171]. Flupirtine is used to treat acute pain in patients resistant to opioids or nonsteroidal anti-inflammatory medicines. Flupirtine has been authorized since the 1980s and is currently available in several countries within the European Union [242]. Currently, Flupirtine has never been investigated in other PH models.

#### 4.3.2. TRPCs and Orais Channels

Overexpression of non-voltage-gated Ca^2+^ channels such as SOCE and ROCE has been implicated in a number of human disorders, including immunodeficiency, autoimmunity, occlusive vascular diseases and cancer, which therefore constitutes an interesting potential therapeutic avenue. Thus, TRPC/Orai could constitute interesting potential targets to treat PAH. Several compounds are available to inhibit their functions.

Imidazole compounds: The imidazole antimycotic SKF-96365 was one of the first identified CRAC (Orai channel family) channel inhibitors. Although this compound inhibits SOCE or ROCE in many cell types. SKF-96365 was shown to suppress the activity of other ion channels, such as voltage-gated-Ca^2+^ channels, nonselective cation channels and cyclic AMP-gated Cl^−^ channels with comparable potencies [243,244], thereby limiting its further clinical use as a specific CRAC/Orai channel modulator.

Diphenylboronate compounds: 2-Aminoethyldiphenyl borate (2-APB) has been widely used to characterize the activity of CRAC. Although 2-APB is widely used to modulate CRAC channel activity, it could also affect K^+^ channels [245], SERCA pumps [246] and mitochondrial Ca^2+^ efflux [247]. Moreover, 2-APB also activates TRPV1, TRPV2 and TRPV3 channels. Mikoshiba’s group identified two 2-APB analogs: DPB162-AE and DPB163-AE. In contrast to 2-APB, DPB162-AE has little effect on TRPC channels, L-type Ca^2+^ channels or Ca^2+^ pump [248] In vivo administration of 2-APB mediates cardioprotection during ischemia reperfusion in mice [249]. 

Pyrazole compounds, the BTPs (bis(trifluoromethyl)pyrazole): A series of BTPs compounds, known as BTP1, BTP2 and BTP3. BTP2 (also known as YM-58483 or Pyr2) does not inhibit voltage-gated-Ca^2+^ or K^+^ channels but activates TRPM4 channels and inhibits the activities of TRPC3 and TRPC5 channels [250]. Three pyrazole derivative compounds, Pyr3, Pyr6 and Pyr10, are described to inhibit Ca^2+^ entry. Pyr3 inhibits both CRAC and TRPC3 channels [251]. Pyr6 displays higher potency inhibition of the CRAC channel than TRPC3, while Pyr10 exhibits significant selectivity for the inhibition of TRPC3 [251]. Interestingly, in vivo application of Pyr3 prevents stent-induced arterial remodeling [252].

In vivo, BTP2 administration attenuates lung ischemia–reperfusion injury in rats [253]. Moreover, in vivo application of BTP2 also prevents the antigen-induced T cell responses that participate in autoimmune diseases [254]. In mice, BTP2 treatment attenuates skin and lungs inflammation, indicating that BTP2-mediated SOCE inhibition may provide some therapeutic interest for immune complex-mediated autoimmunity [255].

Pyrazole compounds, the GSKs: Three other novel pyrazole compounds, GSK5498A, GSK-5503A and GSK-7975A, were identified as selective CRAC channel inhibitors. GSK-5503A and GSK-7975A inhibited STIM1 mediated ORAI1 and ORAI3 currents. Interestingly, these compounds were found to have no effect on many other ion channels [256,257]. Using a murine stroke model, SOCE blockers such as 2-aminoethyl diphenylborate or GSK-7975A suppress platelet-dependent coagulation and thrombus formation [258].

Synta 66: Synta 66, another selective CRAC channel inhibitor [259]. The structure of Synta 66 is similar to Pyr6. This molecule exerts no significant effect on a series of receptors, enzymes and ion channel targets [259,260].

ML204 (4-Methyl-2-(1-piperidinyl)quinolone): TRPC4/C5 are selectively blocked by ML204 without action on TRPV1, TRPV3, TRPA1, and TRPM8, as well as KCNQ2 and native voltage-gated Na^+^, K^+^, and Ca^2+^ channels in mice [261,262].

#### 4.3.3. TRPV Channels

Overexpression of TRPV1 and TRPV4 contribute to the development of PH in the CH-PH rat model. Moreover, TRPV1 was involved in a plethora of physiological and pathophysiological functions [263].

TRPV1 channel could be inhibited by capsazepine. However, in addition to TRPV1 inhibition, capsazepine blocks nicotinic acetylcholine receptors, voltage-gated Ca^2+^ channels and TRPM8.

Regarding the key role played by TRPV1 in in the regulation of pain, several TRPV1 antagonists, including ABT-102, AMG-517, SB-705498, GRC-6211, JNJ-39439335 and MK 2295, were sufficiently safe and effective in preclinical studies to merit testing in clinical trials [263,264]. Nevertheless, side effects are associated with the use of TRPV1 antagonists (regulation of body temperature) [263,264].

Furthermore, in an in vivo asthma model, TRPV1 antagonist, BCTC, attenuated the cough induced by allergens [265].

Highly selective TRPV4 inhibitors, GSK2193874 (hereafter GSK219) [266] and HC067047 [267,268] were shown to be well tolerated in vivo, with minimal side effects. Importantly, oral administration of GSK2193 prevents and resolves pulmonary edema induced by heart failure [266]. Moreover, in vivo inhibition of TRPV4 by HC067047 compounds improves bladder function in mice and rats.

Dysregulation of TRPV1 or TRPV4 channels needs confirmation in human PAH pulmonary vascular cells, and if that is so, TRPV1 and TRPV4 antagonists could be important for PAH.

#### 4.3.4. NMDAR Channels

In vitro, Dumas and colleagues recently found that the noncompetitive NMDAR antagonists MK-801 (dizocilpine) and memantine inhibit human PASMC proliferation, and reverse PH in the MCT-PH rat model [133]. These results indicate that pharmacological inhibition of NMDAR could be considered as a therapeutic strategy in PAH. However, given to the crucial role played by NMDAR in central nervous system, MK-801 treated MCT rats developed central nervous system effects (ataxia, stereotypies, and hyperlocomotion) [133]. Ongoing development of peripheral NMDAR antagonists that do not cross the blood–brain barrier is crucial for the PH field, which should avoid central nervous system adverse effects.

#### 4.3.5. ASIC Channels

Overexpression of ASIC1 in isolated PASMC from CH rats could be an interesting novel target in PAH. In renal tubular epithelial cells, amiloride, a non-specific inhibitor of ASICs, is widely used clinically as a diuretic that inhibits Na^+^ channels [269]. A-317567 is a small molecule (more selective than amiloride) able to block ASIC1a, ASIC2a, and ASIC3 currents [270]. Psalmotoxin (PcTx1) is another selective inhibitor of ASIC1a [271] without effect on currents induced by voltage-gated ion channels, such as some K^+^, Na^+^, and Ca^2+^ channels, and ligand-gated channels [272]. In vivo, selective inhibition of ASIC1a with PcTx1 affords neuroprotection in model of stroke in hypertensive rats [273], and could be an interesting tool for experimental PH models.

#### 4.3.6. TMEM16A Channel

TMEM16A (also called ANO1) expression and function are increased in experimental PH, and could constitute an exciting target for PAH. The channel is inhibited by an aminophenylthiazole, the T16A_inh_-A01, which has never been tested in PH models.

TMEM16A is highly expressed in human cancers for which it represents a critical survival factor. The CaCC_inh_-A01 (ANO1/TMEM16A inhibitor) decreases proliferation [274]. Seo and colleagues found that Ani9 fully block ANO1/TMEM16A channel, without effect on intracellular Ca^2+^ signaling and CFTR chloride channel activity [275].

The small molecule benzbromarone is a well-known inhibitor of TMEM16A [276]. TMEM16A is increased in the airways of asthmatics. A recent study demonstrated its effectiveness in the modulation of mucin secretion and airway smooth muscle contraction, two debilitating features of chronic asthma. These data suggest that TMEM16A could be a unique therapeutic target for asthma, and TMEM16A-CaCC channel blockers, potentially serving as dual-acting agents for the management of asthma.

Chloride Channel Accessory (CLCA) protein family has been recently characterized as regulator of calcium-activated chloride channel currents [277]. CLCA1 expression is associated with airway diseases, thus targeting CLCA represents a promising novel therapeutic pathway to modulate TMEM16/ANO1 channel activity.

Progress is essential to develop more extensive and more selective pharmacological tools that may aid the development of new therapies targeting ion channels in multiple diseases, including PAH.

## 5. Conclusions

**Figure 13:**
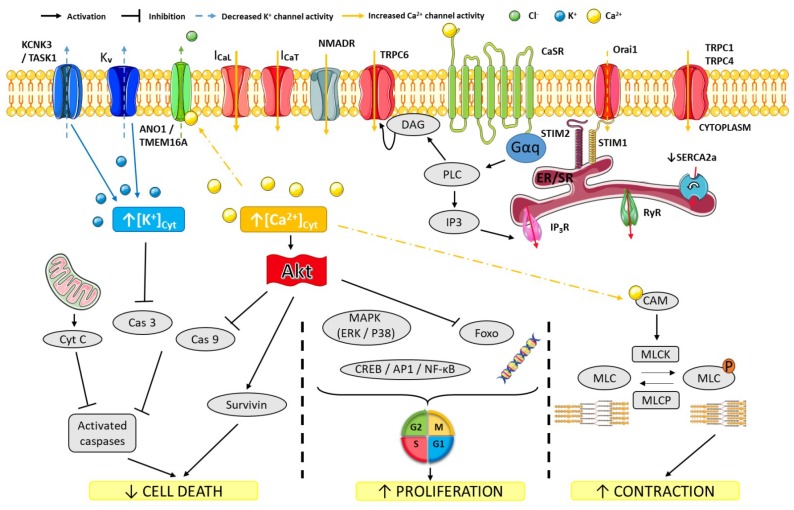
Ion channels remodeling in PAH associated with aberrant cell proliferation, contraction and apoptosis resistance. See description of each channel in the text. TASK-1, TWIK-related acid sensitive K^+^ channel; K_v_, voltage-gated K^+^ channel; TMEM16A, Ca^2+^ sensitive Cl^−^ channel; ICaL, L-type calcium channel; ICaT, T-type calcium channel; TRPC, Transient receptor potential canonical; CaSR, Ca^2+^ sensing receptor; Orai, Calcium release-activated calcium channel protein; STIM1, stromal interaction molecule; SERCA2a, Sarcoplasmic/endoplasmic reticulum Ca^2+^ ATPase; ER/SR, endoplasmic/sarcoplasmic reticulum; IP3R, Inositol 1,4,5 trisphosphate receptor; RyR, ryanodine receptor; CaM, Calmodulin; Cyt C, Cytochrome C; MLC, Myosine light chain; MLCK, Myosine light chain kinase; DAG, Diacylglycerol; PLC, Phospholipase C; IP3, inositol 1,4,5 trisphosphate; NMADR, *N*-methyl-d-aspartate receptor.

PAH represents a major human and social burden because the disease is severe, affecting children and young adults, and the only treatment is lung transplantation for eligible patients which has a high morbidity. Data presented and summarized in Figure 13 suggest that strongly supports ion channels as important players in the pathophysiology of pulmonary vascular diseases and all of them could be carefully considered as relevant novel therapeutic targets for PAH. However, most of them are ubiquitously expressed throughout human tissues. Therefore, this specific pharmacologic effect should be carefully considered in the preclinical development to avoid eventual side effects. Regarding K^+^ channels downregulation in PAH, novel screening approaches such as testing candidate drugs to restore KCNK3/TASK1 or K_v_1.5 channels expressions should be considered. In addition, Cl^−^ channels and non-voltage Ca^2+^ channels might represent novel targets for the treatment of pulmonary vascular diseases as they are upregulated in animal models of PH as well as in pulmonary arteries of idiopathic PAH patients.

## Figures and Tables

**Table 1:** Nomenclature of each K_2P_ channel subunit.

Gene	Channel	Family	Aliases
*KCNK1*	K_2P_1.1	TWIK	TWIK-1
*KCNK2*	K_2P_2.1	TREK	TREK-1
*KCNK3*	K_2P_3.1	TASK	TASK-1
*KCNK4*	K_2P_4.1	TREK	TRAAK
*KCNK5*	K_2P_5.1	TASK	TASK-2
*KCNK6*	K_2P_6.1	TWIK	TWIK-2
*KCNK7*	K_2P_7.1	TWIK	
*KCNK9*	K_2P_9.1	TASK	TASK-3
*KCNK10*	K_2P_10.1	TREK	TREK-2
*KCNK12*	K_2P_12.1	THIK	THIK-2
*KCNK13*	K_2P_13.1	THIK	THIK-1
*KCNK15*	K_2P_15.1	TASK	TASK-5
*KCNK16*	K_2P_16.1	TALK	TALK-1
*KCNK17*	K_2P_17.1	TALK	TALK-2, TASK-4
*KCNK18*	K_2P_18.1		TRIK, TRESK

**Table 2:** Functional impact, changes in the mRNA, protein and function of ion channels reported in PAH and in different models of PH. Upward and downward pointing arrow show increase and decrease, respectively; -, no available information.

Channel	Permeability	mRNA	Protein	Function	Human PAH	Experimental PH Models	Clinical Features
K_v_1.1	K^+^	↓	-	-	Lung from PAH patients		Link to plasma membrane depolarization, to voltage-gated-Ca^2+^ channel activation, to PA vasoconstriction and PASMC proliferation and apoptosis resistance In vivo Kv1.5 re-expression bygene transfert protect against PH in CH rats
		↓	↓	↓		Hypoxic rat PASMC	
K_v_1.2	K^+^	↓	↓	↓		Hypoxic rat PASMC	
K_v_1.5	K^+^	↓	↓	↓	Lung from PAH patients		
		↓	↓	↓		Hypoxic rat PASMC	
		↓	↓	↓		Lung from MCT rat	
K_v_2.1	K^+^	↓	↓	↓		Hypoxic rat PASMC	
		↓	↓	↓		PASMC from CH rat	
K_v_4.3	K^+^	↓	-	-	Lung from PAH patients		
		↓	↓	↓		Hypoxic rat PASMC	
K_v_9.3	K^+^	↓	↓	↓		Hypoxic rat PASMC	
KCNK3/TASK-1	K^+^	-	-	↓	Mutations in PAH patients		Link to plasma membrane depolarization, to voltage-gated-Ca^2+^ channel activation, to PA vasoconstriction and PASMC proliferation and apoptosis resistance. In rat, pharmacological activation with ONO-RS-082 compound reduces MCT phenotype
		↓	-	↓	Lung and PA from iPAH and hPAH patients		
		↓	↓	↓	PASMC from PAH patients	PAEC and PASMC from MCT rat	
		↓	↓	↓		Lung from MCT rat	
BK_Ca_	K^+^	-	↓	↓		PASMC from CH rat	Alteration of PA relaxation
Ca_v_3.1 & Ca_v_3.2	Ca^2+^	-	↑	-	PASMC from iPAH patients		Link to PA vasoconstriction and PASMC proliferation
		↑	↑	-		PASMC from CH rat and mice	
STIM2		-	↑	-	PASMC from iPAH patients		Rat PASMC phenotypic transition
		-	↑	-		Rat PASMC	
Orai2	Ca^2+^	-	↑	-		Rat PASMC	Rat PASMC phenotypic transition and PASMC proliferation
SOCE	Ca^2+^	-	-	↑	PASMC from iPAH patients		
		-	-	↑		Rat distal PASMC	
TRPC6	Ca^2+^, Na^+^	↑	↑	↑	PASMC from iPAH patients		Human and rodents PASMC proliferation In mice *trpc6* gene deletion reduces PH due to CH exposure In human, a polymorphism in *TRPC6* gene seems to predispose to PAH
		↑	↑	-	Lung and PASMC from iPAH patients		
		-	↑	-		Milan Hypertensive rats	
		↑	↑	-		Lung from CH rats	
TRPC1	Ca^2+^, Na^+^	↑	-	-		PASMC from CH mice	Rodents PASMC proliferation In mice *trpc1* gene deletion reduces PH due to CH exposure
		↑	↑	↑		PASMC from CH / MCT rats	
TRPC3	Ca^2+^, Na^+^	↑	↑	↑	PASMC from iPAH patients		
TRPV4	Ca^2+^, Na^+^	↑	↑	↑		PASMC from CH rat	PASMC migration and proliferation In mice, *trpv4* gene deletion suppresses the development of PH due to CH
TRPV1	Ca^2+^, Na^+^	↑	↑	-	PASMC from iPAH patients		PASMC proliferation and migration.
ASIC	Na^+^, Ca^2+^	-	↑	↑		PASMC from CH rat	Link to membrane depolarization and rat PA vasconstriction
CaSR	Ca^2+^	-	↑	↑	PASMC from iPAH patients		PASMC proliferation
SERCA2a	Ca^2+^	-	↓	-	Lung and PASMC from iPAH patients	Lung and PASMC from MCT rats	PASMC proliferation and migration. *In vivo* SERCA2a re-expression by gene therapy reduce MCT-PH phenotype
SCN1B	Na^+^	↑	-	↑	Lung from iPAH patients		Contribute to abnormal vasoconstriction and pulmonary vascular remodeling
NMDAR	Ca^2+^	↑	↑	-	PA from iPAH patients		Link to PASMC proliferation, In rat NMDAR antagonist reduce PH, In mice, *nmdar* gene deletion reduces PH
TMEM16A	Cl^-^	↑	↑	↑		Lung from MCT rat	Link to membrane depolarization and rat PA vasconstriction
		↑	↑	-		Lung from CH rat	

**Table 3:** Clinical measurements at diagnosis of *KCNK3* mutation carrier PAH patients (Ma et al., 2013 [11]; Higasa et al. 2017 [15]; Navas tejedor et al., 2017 [13]; Best et al., 2017 [14]) mPAP, mean pulmonary arterial pressure; RAP, Right atrial pressure; CI, Cardiac index; PVI, pulmonary vascular resistance index.

		All	Females	Males
Age of Diagnosis	Median	29	35	22
	Max	52	48	52
	Min	0.17	17	0.17
mPAP (mmHg)	Median	76	67	94
	Max	107	81	107
	Min	54	54	86
RAP (mmHg)	Median	12	15.5	11
	Max	29	29	16
	Min	3	7	3
CI (L/min)	Median	2.7	2.7	2.36
	Max	3.22	3.16	3.22
	Min	1.21	1.21	1.73
PVI (dyn/s/cm^−5^)	Median	1826	1764	2874
	Max	3977	3174	3977
	Min	1316	1316	1813
